# Heteroelement Analogues of Benzoxaborole and Related Ring Expanded Systems

**DOI:** 10.3390/molecules26185464

**Published:** 2021-09-08

**Authors:** Krzysztof Nowicki, Piotr Pacholak, Sergiusz Luliński

**Affiliations:** 1 Faculty of Chemistry, Warsaw University of Technology, Noakowskiego 3, PL-00-664 Warsaw, Poland; krzysztof.nowicki2.dokt@pw.edu.pl (K.N.); piotr.pacholak94@gmail.com (P.P.); 2 Faculty of Chemistry, University of Warsaw, Pasteura 1, PL-02-093 Warsaw, Poland

**Keywords:** boron, heterocycles, benzoxaborole, Lewis acidity, antimicrobial activity

## Abstract

The review covers the chemistry of organoboron heterocycles structurally related to benzoxaboroles where one of the carbon atoms in a boracycle or a fused benzene ring is replaced by a heteroelement such as boron, silicon, tin, nitrogen, phosphorus, or iodine. Related ring expanded systems including those based on naphthalene and biphenyl cores are also described. The information on synthetic methodology as well as the basic structural and physicochemical characteristics of these emerging heterocycles is complemented by a presentation of their potential applications in organic synthesis and medicinal chemistry, the latter aspect being mostly focused on the promising antimicrobial activity of selected compounds.

## 1. Introduction

**Scheme 1:**
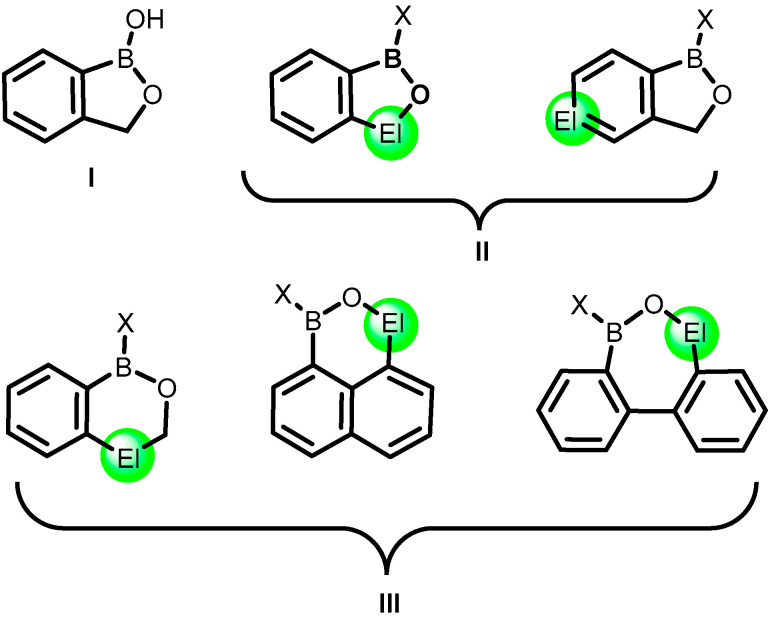
Structures of benzoxaborole, I; its heteroelement congeners, II; and related ring-expanded systems, III. El stands for heteroatom or heteroatom-based fragment.

Recently, benzoxaboroles (Scheme 1, structure I) constitute one of the leading groups of organoboron compounds. This is mainly due to their promising biological properties, which have been exploited for the past 20 years in medicinal and bioanalytical chemistry [1,2,3,4]. Benzoxaboroles are strongly predestined for such applications due to their improved thermodynamic stability, resulting from the presence of a strong covalent boron-oxygen bond. Overall, they are rather stable to air and water and, in general, do not undergo rapid degradation under in vivo conditions. Therefore, heteroelement analogues of benzoxaboroles (Scheme 1, general structures II) constitute an interesting alternative and may offer the opportunity for various novel applications while retaining high stability arising from the presence of a strong B-O bond in the ring structure. The replacement of a carbon atom in the boracycle or an adjacent benzene ring with a different atom may result in a significant change of structural behaviour, e.g., a tendency to aggregation involving dative interactions of a heteroatom with the boron atom. Moreover, the presence of a heteroatom may result in modified physicochemical properties, including solubility, lipophilicity, hydrolytic stability, boron Lewis acidity, and others. The aim of this review is to highlight several emerging groups of boracyclic systems which comprise various heteroelement atoms such as another boron, silicon, tin, nitrogen, phosphorus, and iodine. Some ring expanded analogues (Scheme 1, general structures III), including compounds based on naphthalene and biphenyl-scaffold, are also included. Overall, the review is divided into sections based on type of heteroelement and heterocyclic ring as the primary and secondary classification criteria, respectively. The synthesis and physicochemical properties as well as applications of compounds of interest are consecutively presented in each section.

## 2. Benzoxadiboroles and Related Ring-Expanded Systems Comprising B-O-B Linkage

**Scheme 2:**
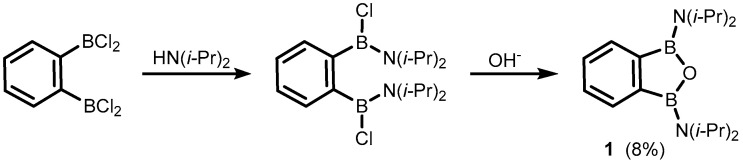
Synthesis of 1,3-bis(diisopropylamino)-1,3-dihydro-2,1,3-benzooxadiborole.

The formal substitution of the C3 carbon atom in benzoxaborole results in a benzoxadiborole framework featuring a B-O-B linkage within the five-membered ring. An example of such a well-defined boracyclic system (**1**) was reported by Kaufmann et al. in 1994 [5]. It was isolated in a low yield by aminolysis of 1,2-bis(dichloroboryl)benzene [6] followed by ring closure with hydroxide anion (Scheme 2).

**Scheme 3:**
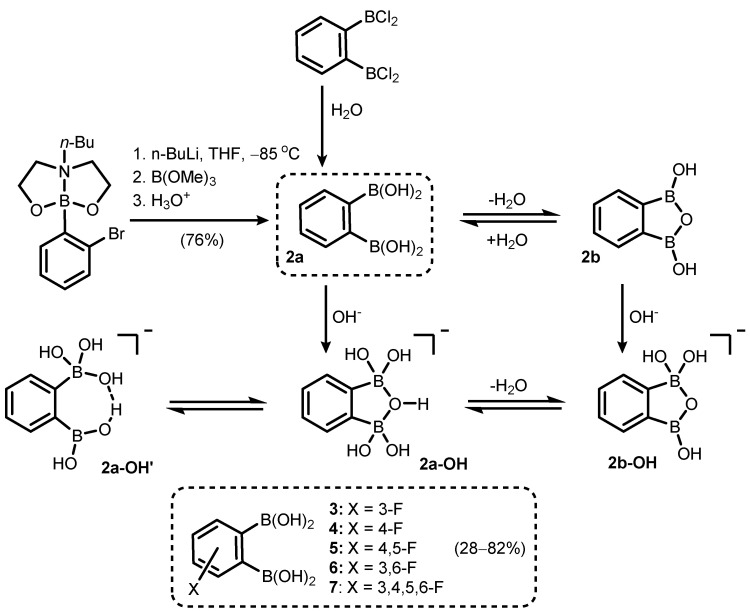
Synthesis of phenylene-1,2-diboronic acid (**2a**). Cyclization and acid-base equilibria involving **2a** and 1,3-dihydroxybenzoxadiborole (**2b**).

Phenylene-1,2-diboronic acid (**2a**) was found to be a useful precursor of benzooxadiborole derivatives (Scheme 3). It can be readily obtained by careful hydrolysis of 1,2-bis(dichloroboryl)benzene [6] or a Br/Li exchange reaction of 2-(2-bromophenyl)butyl[1,3,6,2]dioxazaborocan, followed by quenching the aryllithium intermediate with B(OMe)_3_ (Scheme 3) [7]. Subsequent studies on the structural behaviour of **2** and its fluorinated derivatives (**3**–**7**) revealed that those compounds tend to equilibrate in solution with respective cyclic semi-anhydrides, i.e., 1,3-dihydroxy-1,3-dihydro-2,1,3-benzoxadiboroles (Scheme 3). ^1^H and ^13^C NMR analyses in various dry deuterated solvents (acetone, THF, DMSO) revealed that cyclization (**2a**→**2b**) occurs to a significant extent. Moreover, the ^11^B NMR spectrum showed that boron atoms (**2**) are slightly deshielded (by ca. 4 ppm) with respect to free acid. However, the B-O-B linkage is readily cleaved upon the addition of water (or D_2_O), shifting the equilibrium towards free acid (**2b**).

The formation of 1,3-dihydroxybenzoxadiborole scaffold (**2b**) (Scheme 3) clearly accounts for the apparent stronger acidity (p*K*_a_ = 6.0) of the entire equilibrium system compared to related acyclic meta- and para-substituted phenylenediboronic acids [8,9]. Theoretical (DFT B3LYP) studies indicate that the relative stabilization the anionic form (**2b-OH**) is important in this respect, although the persistence of its hydrated forms, i.e., a cyclic species (**2a-OH**) with a bridging hydroxyl anion bound simultaneously by two boronic groups in a bidentate fashion and an unsymmetrical form (**2a-OH′**), stabilized by charge-assisted intramolecular H-bond, should also be taken into account.

**Scheme 4:**
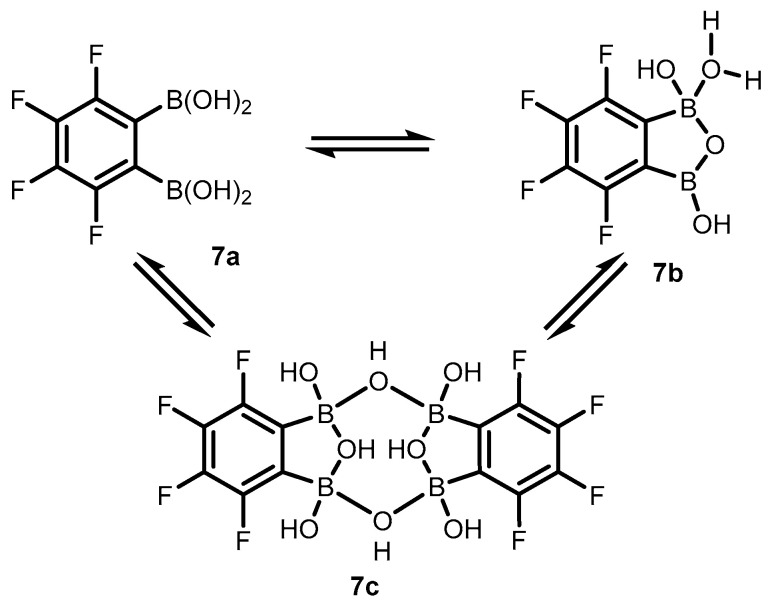
Structural diversity of 3,4,5,6-tetrafluorophenylene-1,2-diboronic acid (**7**) involving the standard open form (**7a**), the benzoxadiborole tautomer (**7b**) and the cyclic dimer (**7c**).

Subsequent studies revealed that 3,4,5,6-tetrafluorophenylene-1,2-diboronic acid **7** shows a stronger tendency to intramolecular cyclization. X-ray diffraction analysis confirmed the formation of perfluorinated benzoxadiborole (**7b**), complexed with water molecules (Scheme 4) [8]. Interestingly, a unique dimeric form of **7c** was also obtained by crystallization in toluene. The molecule consists of two benzoxadiborole frameworks fused by means of two B-OH-B bridges and additionally stabilized by π-π interactions of aromatic rings, resulting in a general chair-type conformation. Overall, the impact of perfluorination results in the strong acidity enhancement of **7** compared to **2**, leading to an apparent p*K*_a_ of 3.0, which is among the lowest figures for boronic acids and related species.

**Scheme 5:**
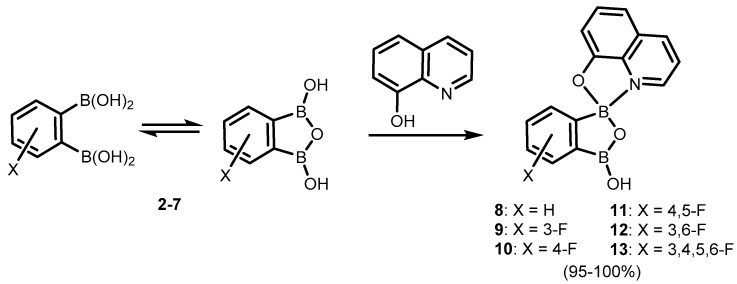
Synthesis of benzoxadiborole 8-oxyquinolinato complexes **8**–**13**.

**Scheme 6:**
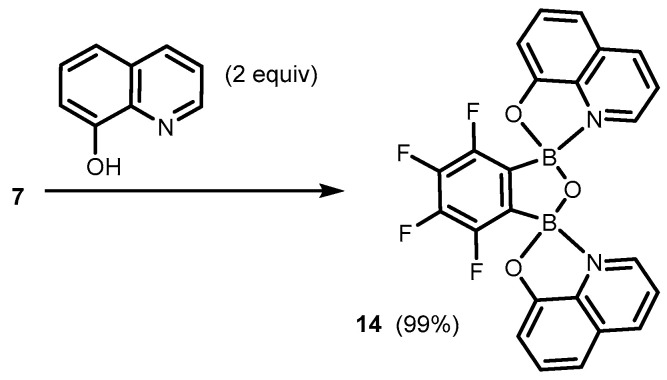
Synthesis of benzoxadiborole bis(8-oxyquinolinato) complex **14**.

**Scheme 7:**
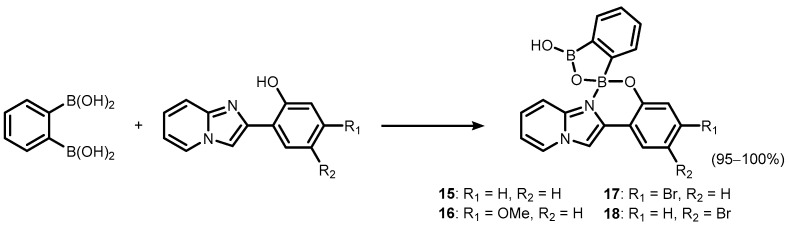
Synthesis of benzoxadiborole (*O*,*N*)-chelate complexes **15**–**18** with 2-(imidazo[1,2-a]pyridin-2-yl)phenol.

It was found that the benzoxadiborole scaffold is strongly stabilized upon treatment with -8-hydroxyquinoline (Scheme 5) [10]. The reactions of **2a/2b** and its fluorinated derivatives **3**–**7** afforded respective chelate complexes **8**–**13**, both in solution and under mechanochemical conditions. The most Lewis acidic **7** also bound readily two 8-oxyquinolinato ligands, yielding bis(chelate) (**14**) (Scheme 6) [10]. All of the obtained complexes exhibit green luminescence in acetonitrile solution (λ_em_ = ca. 525 nm, Φ = 13–15%), resembling other organoboron 8-oxyquinolinato complexes. Interestingly, it is blue-shifted in solid state (λ_em_ = ca. 500 nm), which was ascribed to the effect of H-bonding and other polar interactions of discrete molecules in the crystal lattice. Importantly, the electroluminescence properties of complexes **8** and **14** was proved by testing OLEDs containing those compounds as emitters [10]. Later on, complex **8** became the subject of in-depth structural characterization, which included interesting solvatomorphic behaviour [11] as well as high resolution single-crystal X-ray diffraction electron density studies performed for the first time in the case of a luminescent oxyquinolinato organoboron complex [12]. Furthermore, **2** was also employed for the synthesis of a series of luminescent (*O*,*N*)-chelate complexes (**15**–**18**) with 2-(imidazo[1,2-a]pyridin-2-yl)phenol ligands (Scheme 7) [13]. The products were also characterized by single crystal X-ray diffraction, which revealed formation of H-bonded dimers in the solid state.

**Scheme 8:**
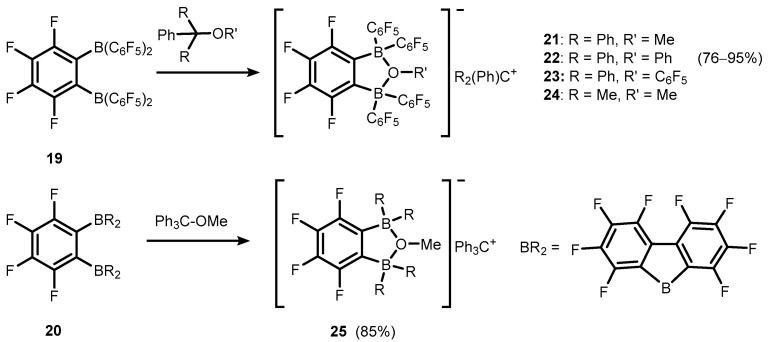
Synthesis of ion-pair compounds **21**–**25**, comprising tertiary carbocations and weakly-coordinating anions based on perfluorinated benzoxadiborole backbones.

**Scheme 9:**
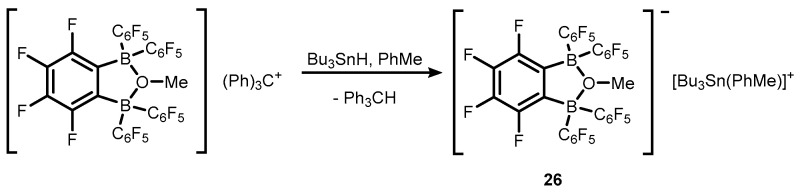
Synthesis of stannylium cationic species **26**.

Transformations of strong bidentate Lewis acids of a general formula *o*-C6F4(BR2)2, R = C_6_F_5_ (**19**), and BR_2_ = BC_12_F_8_ (**20**) gave rise to various anionic or neutral boracyclic species structurally closely related to **7b**/**7c** (Scheme 8) [14,15,16,17]. However, it should be noted that most of them are formed, at least in a formal sense, by means of dative O→B interactions. Specifically, weakly-coordinating borate anions *o*-C6F4[B(C_6_F_5_)2]2(*μ*-OR), R = Me, Ph, C_6_F_5_, and C6F4[BC_12_F_8_]2(*μ*-OMe) were employed for stabilization of selected tertiary carbocations in respective ion-pair compounds (**21**–**24** and **25**, respectively) (Scheme 8) [15,16,17,18]. It was found that trityl salts (**21**–**23**) are effective co-catalysts of ethylene polymerization due to activation of dimethyl zirconocene (Cp_2_ZrMe_2_), resulting in corresponding products with Cp_2_ZrMe^+^ cation [15,18]. Compound **21** was also used for generation of stannylium cationic species **26** (Scheme 9) [19].

**Scheme 10:**
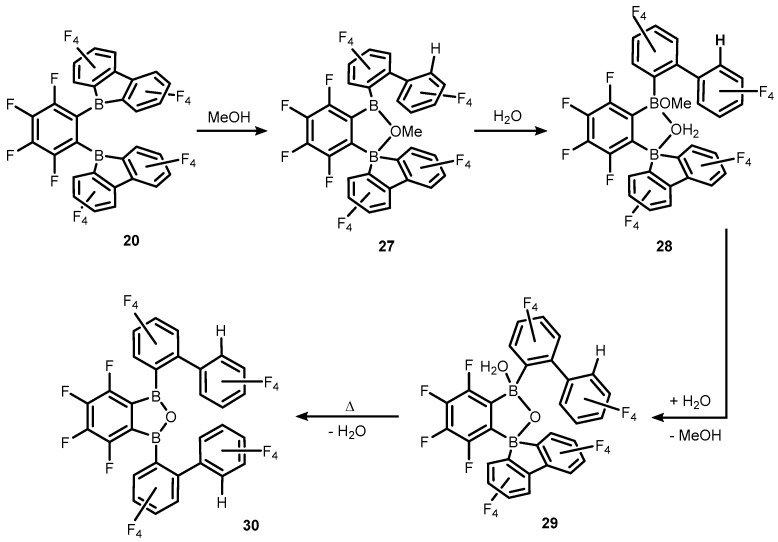
Transformations of **20** to various boracyclic systems (**27**–**30**) upon interactions with MeOH and H_2_O.

The analogous oxonium salt with (Et_2_O)_2_H^+^ counterion was also obtained [19]. Similarly, related Bronsted acids based on solvated protons were generated from reactions of **19**–**20** with an excess of protic reagents (MeOH, H_2_O) (Scheme 10) [20]. It should be noted that such species are generally prone to protolytic cleavage of B-C, which results in fragmentation of a boracyclic anions derived from **19**. On the other hand, controlled treatment of **20** with MeOH/H_2_O gives rise to various neutral species such as cyclic borinic ester (**27**) obtained upon protonolysis of one B-C bond in the borafluorene ligand, the unique system (**28**) with water molecule bridging two boron centres, water-coordinated borinic acid (**29**) as well as the benzoxadiborole (**30**) arising from the cleavage of another B-C bond. The molecular structures of compounds **27**–**30** were determined by X-ray diffraction. The studies on the reactivity of **19**–**20** towards water were directly connected to their use as potent initiators of isobutene polymerization. They were aimed at shedding light on the plausible role of dissolved water as a chain transfer agent in polymerizations involving **19**–**20** that give rise to weakly-coordinating counteranions. It was suggested that species featuring bridging water molecule such as compound **28** are active as a strong Brønsted acid that is able to protonate isobutene which initiates the polymer chain growth [20].

**Scheme 11:**
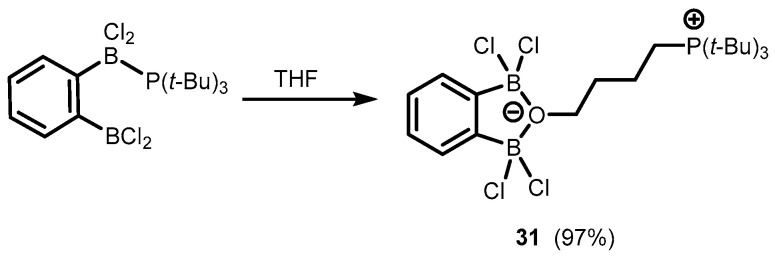
Synthesis of a zwitterionic system **31** based on an anionic benzoxadiborole framework.

In addition, one can also mention herein the synthesis of a zwitterionic system (**31**) based on an anionic benzoxadiborole framework with a C_4_-chain attached to an oxygen atom and decorated with a cationic phosphonium end group. This was obtained by the ring opening of the THF molecule due to interaction with a Frustrated Lewis Pair system composed of 1,2-bis(dichloroboryl)benzene and tris(*tert*-butyl)phosphine (Scheme 11) [21].

**Scheme 12:**
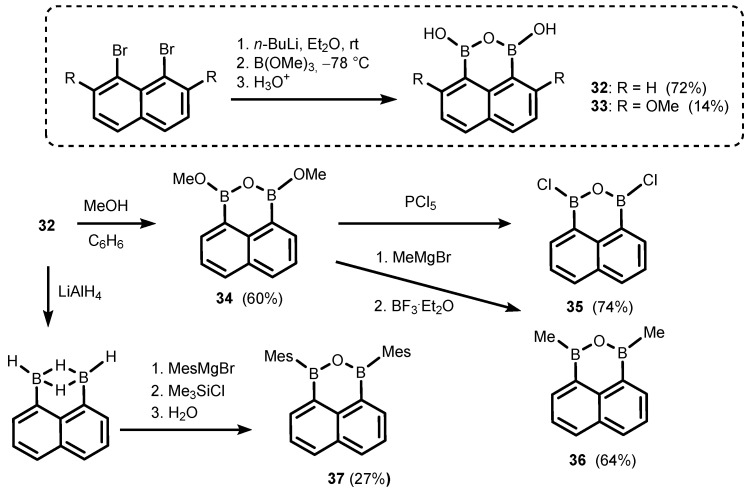
Synthesis of 1,3-dihydroxy-1*H*,3*H*-naphth[l,8-*cd*][l,2,6]oxadiborins (**32**–**33**) and conversion of **32** to **34**–**37**.

The ring-expanded benzoxadiborole analogues are based on naphthalene and biphenyl scaffolds. Thus, 1,3-dihydroxy-1*H*,3*H*-naphth[l,8-*cd*][l,2,6]oxadiborin (**32**) (Scheme 12) is easily accessible by diboronation of 1,8-dilithionaphthalene with B(OMe)_3_ [22,23,24]; 4,9-Dimethoxy derivative (**33**) was obtained analogously and characterized by single-crystal X-ray diffraction [25]. Compound **32** was successfully used as a coupling partner in selected Suzuki–Miayura cross-coupling reactions, resulting in the formation of new aryl-aryl bonds [26,27,28]. Thus, its behaviour seems to be rather typical of arylboronic acids and their derivatives. However, unlike benzoxadiboroles, the B-O-B linkage in **32** seems to be rather stable as there are no data which might indicate that a reversible hydrolysis to naphthalene-1,8-diboronic acid occurs to any appreciable extent. Compound **32** was used as a starting material for synthesis of a few 1*H*,3*H*-naphth[l,8-*cd*][l,2,6]oxadiborin derivatives (**34**–**37**) where hydroxyl groups were replaced with OMe, Cl, Me [23], or mesityl (Mes) substituents [24], respectively.

**Scheme 13:**
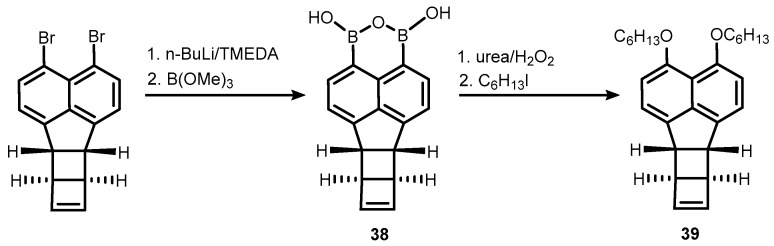
Synthesis and transformation of a boracyclic system (**38**) featuring B-O-B linkage attached to bicyclohexene-peri-naphthalene scaffold.

Very recently, a structurally extended analogue of **32** based on bicyclohexene-*peri*-naphthalene framework **38** was obtained using an analogous protocol involving bromine-lithium exchange in an appropriate dibromide, followed by boronation. It should be stressed that the system features a significant ring strain arising from the presence of two C4 and one C5-ring fused with the naphthalene core. Nevertheless, **38** was used successfully for the synthesis of peri-substituted bis(hexyl ether) (**39**)via oxidation of B-C bonds followed by alkylation (Scheme 13) [29].

**Scheme 14:**
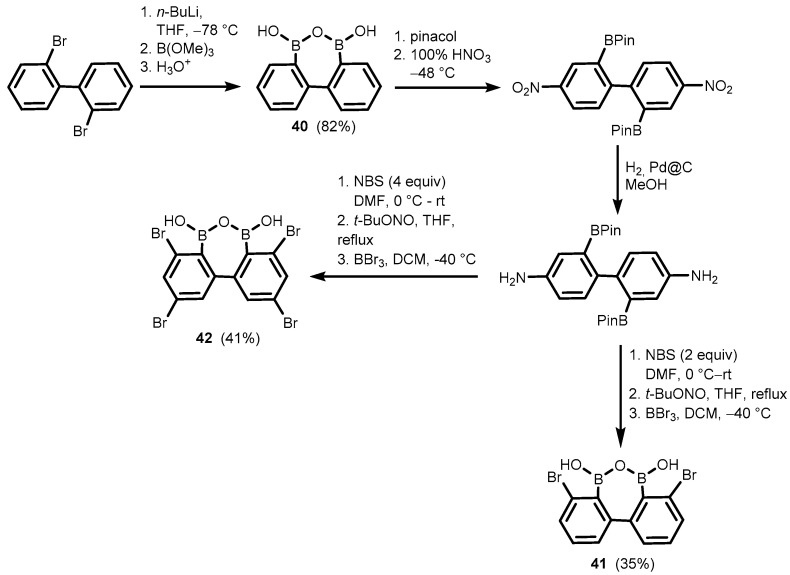
Synthesis of oxadiborepins **40**–**42**.

**Scheme 15:**
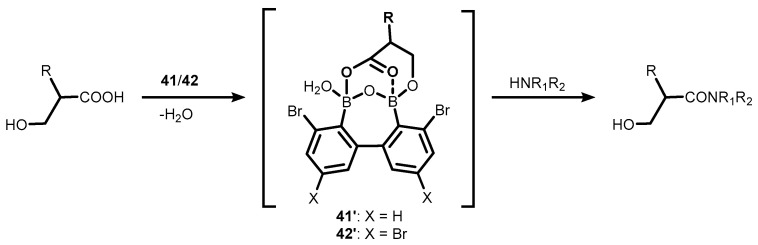
Direct amidation of β-hydroxy substituted carboxylic acids with amines in the presence of bromo-substituted oxadiborepins **41**–**42**.

Related oxadiborepins, i.e., 7-membered boracyclic systems comprising B-O-B linkage and 2,2′-biphenyl core, were also obtained [30,31,32]. It should be noted that the plausible equilibrium between biphenyl-2,2′-diboronic acid and its cyclic semi-anhydride (**40**) has not been studied to date, although its synthesis was reported already in 2002 [30], followed by crystallographic determination of **38** in 2011 [31]. Compounds **41**–**42** were obtained in a multistep protocol starting with 2,2′-dibromobiphenyl (Scheme 14). Remarkably, **41**–**42** were reported as efficient catalysts of dehydrative amidation of carboxylic acids with amine substrates. Initially, they were employed for efficient preparation of various α- and β-hydroxy substituted amides [32] but thereafter also proved effective in catalyzing the formation of Weinreb amides [33,34] as well as various oligopeptides [35]. In the former case, the proposed mechanism of the catalytic process involves the cooperation of the two boron atoms in **41**–**42**, which enables the formation of a cyclic mixed anhydride with carboxylic acid molecule, as evidenced by the ESI MS spectrum; this is followed by an attack of amine on the activated carbonyl group (Scheme 15) [32]. It should be noted that the performance of **42** is impressive, as evidenced by low catalyst loading (even a 0.01 mol% turnover number (TON) parameter up to 7500).

**Scheme 16:**
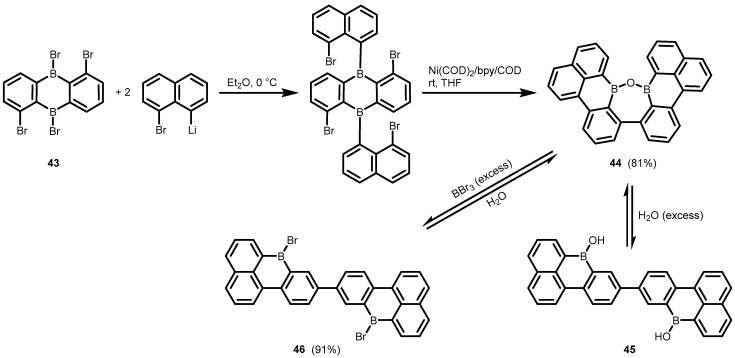
Synthesis and cleavage of the fused polycyclic oxadiborepin **44**.

One can also mention the unexpected synthesis of the fused polycyclic oxadiborepin (**44**) from the diboraanthracene precusor (**43**) which involved double arylation with 8-bromo-1-naphthyllithium followed by successful debromination/C–C coupling using Ni(COD)_2_/bpycatalyst (Scheme 16) [36]. The formation of a third C–C bond and the cleavage of two B–C bonds was observed when THF was used as the solvent. The ^1^H NMR studies on the structural behaviour of **44** revealed that it exists in equilibrium with the respective diborinic acid (**45**) upon the addition of water, whereas complete conversion to the dibromo derivative (**46**) occurs upon heating with an excess of BBr**_3_**. Compound **46** is readily reconverted back to **44** upon the addition of water.

## 3. Benzosiloxaboroles and Related Ring-Expanded Systems Comprising B-O-Si Linkage

**Scheme 17:**
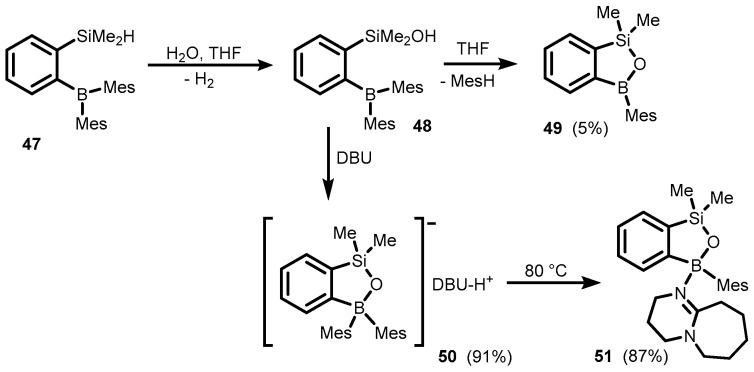
Synthesis of benzosiloxaborole derivatives through intramolecular cleavage of B-C bond with SiOH group.

Benzosiloxaboroles are silicon analogues of benzoxaboroles where the carbon atom in a boracyclic ring is replaced by a silicon atom, thus resulting in the formation of a B-O-Si linkage. The first benzosiloxaborole derivative bearing mesityl group at the boron atom (**49**) was synthesized in 2008 (Scheme 17) [37,38]. The starting 1-dimesitylboryl-2-(dimethylsilyl) benzene (**47**) was subjected to hydrolysis of the Si-H bond followed by ring closure effected through an attack of silanol on one of the B-Mes bonds, yielding mesitylene as a byproduct. The reaction occurs slowly in THF at rt, but can be accelerated by heating or the addition of tertiary amine such as Et_3_N and diazabicyclo[5.4.0]undec-7-ene (DBU). In the case of the latter, one can isolate an intermediate ionic compound (**50**) with the boracyclic anion resulting from the quantitative deprotonation of silanol (**48**). When heated in THF at 80 °C, it undergoes transformation to complex **51** with the DBU ligand coordinated to the boron atom in **49**.

**Scheme 18:**
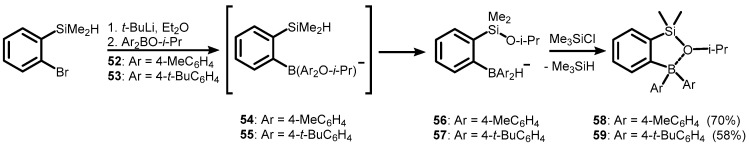
Synthesis of ortho-(alkoxysilyl) arylboranes (**58**–**59**) featuring dative Si-O(*i*-Pr)→B linkage.

The bromine-lithium exchange reaction of *ortho*-(dimethylsilyl)bromobenzene with *t*-BuLi, followed by trapping with (isopropoxy)diarylboranes (**52**–**53**), resulted in borohydride intermediates (**56**–**57**), which form neutral *ortho*-(alkoxysilyl)(diarylboryl)benzenes (**58**–**59**) after the addition of chlorotrimethylsilane. The formation of **56**–**57** can be explained in terms of intramolecular hydride–isopropoxide exchange between silicon and boron atoms in initially formed unstable alkoxyborate complexes (**54**–**55**) (Scheme 18) [39].

**Scheme 19:**
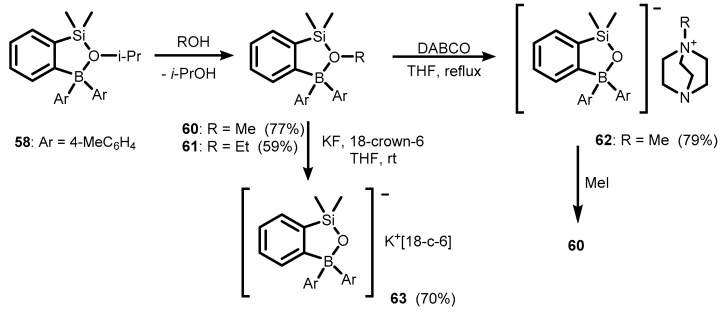
Synthesis of ionic compounds **62**–**63**, featuring benzosiloxaborolate anion.

Compound **58** reacts readily with MeOH and EtOH to give methoxysilane **60** and ethoxysilane **61**, respectively (Scheme 19) [39]. The ^11^B and ^29^Si NMR spectroscopy data of **58**–**61** point to intramolecular O→B coordination, which results in the existence of cyclic structures. In addition, X-ray diffraction analysis of **60** confirmed the formation of a boracyclic ring involving a rather strong dative Si-O(Me)→B linkage (B-O distance of 1.652(2) Å). Remarkably, this interaction enhances reactivity of the Me-O bond owing to the enhanced electrophilic character of the carbon atom. Thus, the treatment of **60** with 1,4-diazabicyclo[2.2.2]octane (DABCO) afforded ionic complex **62**, composed of silyloxyborate anion and DABCO-Me cation (Scheme 19). However, the oxygen atom in the anion is nucleophilic and can be readily methylated to regenerate **60**. Finally, the cleavage of the Me-O bond in **60** can also be performed using KF in the presence of 18-crown-6 ligand to give potassium salt (**63**).

**Scheme 20:**
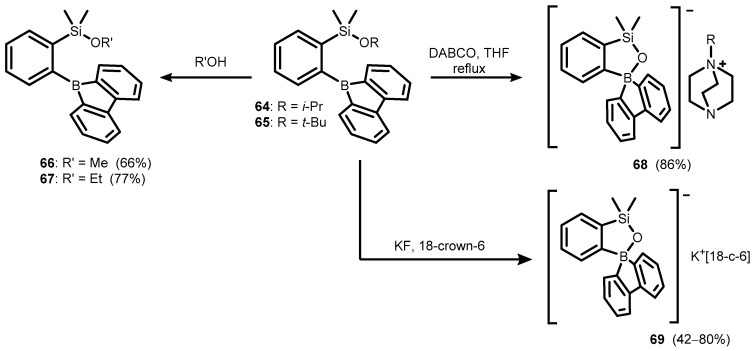
Synthesis of ionic compounds **68**–**69** featuring benzosiloxaborolate anion comprising 9-borafluorenyl moiety.

Analogous *ortho*-(alkoxysilyl)(borafluorenyl)benzenes (**64**–**65**) were obtained using the protocol described above for **58**–**59**. Compound **64** was readily converted to respective methoxy- and ethoxysilanes (**66**–**67**) and resulted in ionic compounds **68**–**69** upon treatment with 1,4-diazabicyclo[2.2.2]octane (DABCO) and KF/18-crown-6, respectively (Scheme 20) [40].

**Scheme 21:**
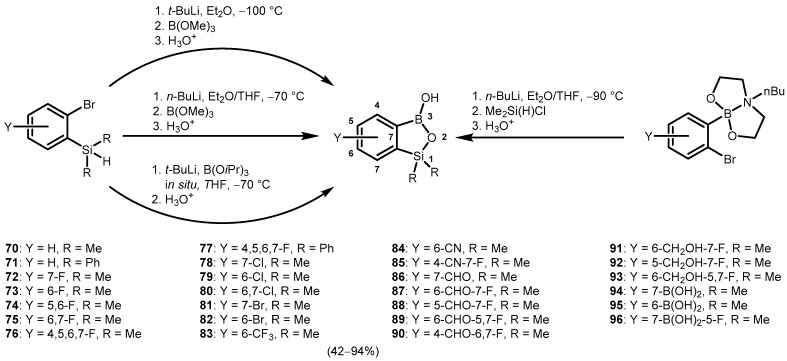
Synthesis of functionalized benzosiloxaborole derivatives.

The synthesis of a benzosiloxaboroles bearing hydroxy group at the boron atom, i.e., 3-hydroxybenzosiloxaboroles, was accomplished using two general protocols. The first one involves bromine-lithium exchange in ortho-bromoaryl(dimethyl)silanes and subsequent boronation with trialkyl borates B(OR)_3_ (R = Me, Et, *i*-Pr), followed by hydrolysis, leading to intramolecular dehydrogenative condensation (Scheme 21) [41,42,43]. The second method involves the addition of Me_2_Si(H)Cl to 2-(2-lithiophenyl)-butyl-1,3,6,2-dioxazaborocan or its functionalized analogue, generated via Br-Li exchange using *n*-BuLi in Et_2_O/THF (1:1) at low temperatures, typically below −90 °C (Scheme 21) [41,42,43]. Extensive work resulted in the preparation of a number of 3-hydroxybenzosiloxaboroles (**70**–**96**) bearing substituents such as F, Cl, Br, CF_3_, CN, CHO, CH_2_OH, and B(OH)_2_, attached at various positions of the aromatic ring [41,42,43].

**Scheme 22:**
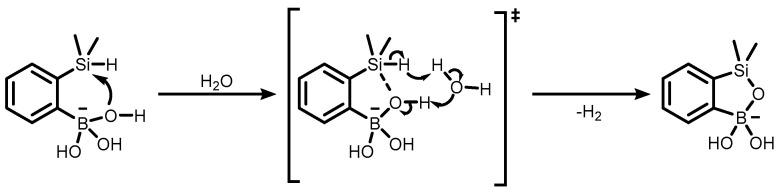
The proposed mechanism for the activation of the Si–H bond by the anionic boronate group in the presence of water.

The formation of 3-hydroxybenzosiloxaboroles was the subject of some mechanistic studies aimed mainly at the elucidation of the activation pathways of the Si-H bond in boronated arylsilane precursors. DFT (M06-2X22/6-31+G(d)23) theoretical calculations revealed that the process of ring closing is driven by the coordination of an oxygen atom from the B(OH)_2_ or, far better, from the anionic B(OH)_3_^−^ group to the silicon atom (Scheme 22) [44].

**Scheme 23:**
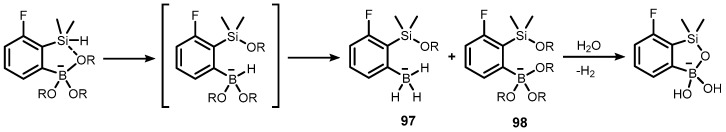
Activation of the Si–H bond by the anionic trialkoxyboronate group through hydride-alkoxy exchange between silicon and boron [41].

On the other hand, experimental studies showed that, in the absence of water, the ortho-(dimethylsilyl)-substituted trialkoxyaryl(boronate) anion undergoes hydride transfer from silicon to boron to give a mixture of tris(hydrido)arylborate and tris(alkoxy)arylborate anions (**97**–**98**) as confirmed by ^11^B NMR spectroscopy data (Scheme 23) [43]. Hydrolysis of the mixture proceeds with hydrogen evolution, and the final intramolecular condensation of transiently generated silanol and boronic groups occurs readily to give the siloxaborole ring. In fact, DFT calculations indicate that this process is thermodynamic and favourable, to a similar extent, as the condensation of carbinol and boronic groups, resulting in the formation of B-O-C linkage in benzoxaboroles [41].

**Scheme 24:**
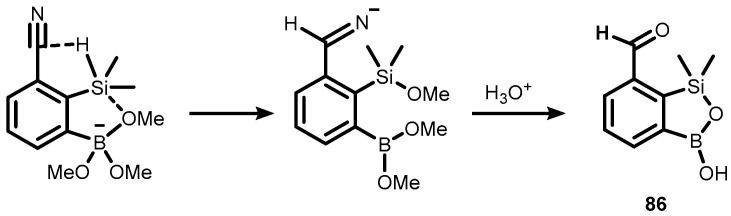
Activation of the Si–H bond by the anionic trialkoxyboronate group, resulting in reduction of the nitrile group and formation of (**86**).

It was found that the activated Si-H bond was able to reduce nitrile to the formyl group, which was used for synthesis of compound **86** (Scheme 24) [42].

**Scheme 25:**
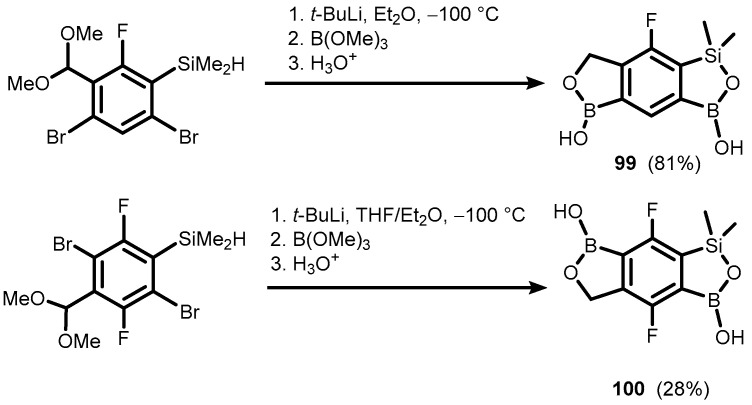
Synthesis of **99**–**100** involving the reduction of acetal CH(OMe)_2_ group with activated Si–H bond.

**Scheme 26:**
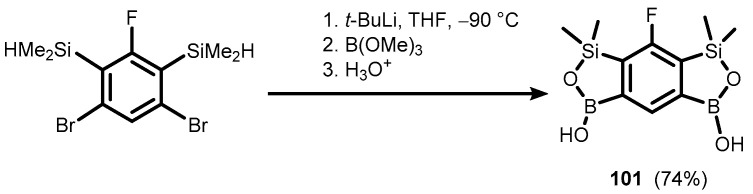
Synthesis of benzobis(siloxaborole) **101**.

Interestingly, the reduction of otherwise rather inert acetal CH(OMe)_2_ groups was observed, giving rise to benzosiloxaboroles comprising an additional fused oxaborole ring (**99** and **100**) (Scheme 25) [42]. An analogous system (**101**) with two siloxaborole heterocycles fused with the central aromatic ring was also prepared using a double Br-Li exchange in appropriate bis(dimethylsilyl)dibromobenzene, followed by boronation (Scheme 26) [43].

**Scheme 27:**
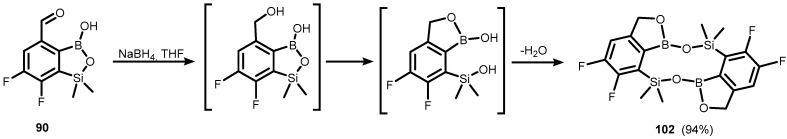
Synthesis of extended borasiloxane heterocycle system (**102**) through the reduction of **89**.

It should be noted that formyl-substituted benzosiloxaboroles (**87**–**89**) are cleanly reduced to respective carbinol derivatives (**91**–**93**) using NaBH_4_, whilst an analogous reaction of **90** afforded a unique dimeric species (**102**) featuring a central 10-membered heterocycle comprising two B-O-Si linkages. Its formation is apparently driven by the preferred formation of an oxaborole ring with concomitant breakdown of the siloxaborole ring with the release of free silanol moiety. Subsequent bimolecular dehydrative condensation leads to the formation of **102** (Scheme 27) [42].

**Scheme 28:**
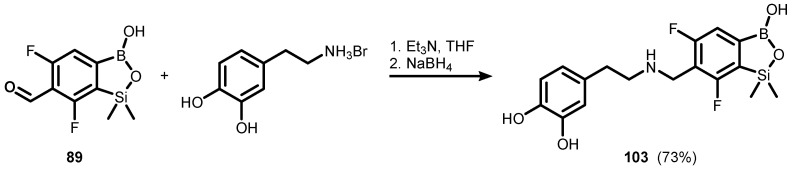
Reductive amination of (**89**) leading to dopamine conjugate (**103**).

In addition, a formyl derivative (**89**) was successfully subjected to reductive amination with dopamine, giving rise to the benzosiloxaborole conjugate (**103**) (Scheme 28) [42].

**Scheme 29:**
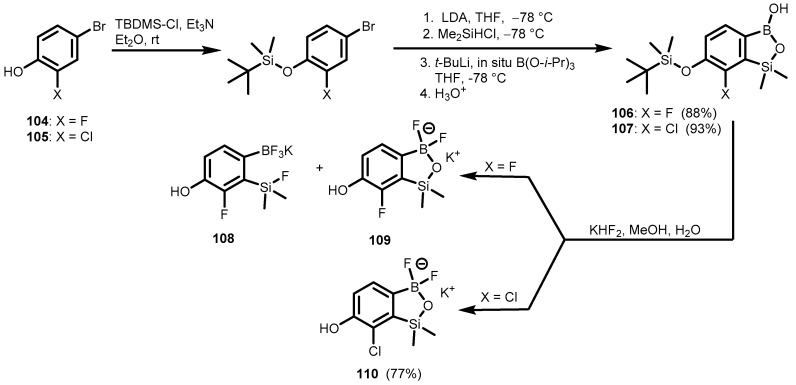
Synthesis of potassium hydroxy-substituted benzosiloxa(difluoro)borolates **109**–**110**.

Very recently, the 6-hydroxy-7-chloro substituted 3,3-difluorobenzosiloxaborolate anion was prepared in the form of a potassium salt (**110**), starting with the protection of 4-bromo-2-chlorophenol (**105**) with tert-butylsilyldimethyl chloride (TBDMS-Cl), followed by introduction of the Si(H)Me_2_ group, subsequent boronation, and cleavage of the silyl ether group (OTBDMS) in benzosiloxaborole (**107**) (Scheme 29) [45]. Similar reactions were performed using 4-bromo-2-fluorophenol (**104**) as the starting material. Unfortunately, the final cleavage of the OTBDMS group in **106** resulted in a mixture containing a significant amount of aryltrifluoroborate salt (**108**), together with the desired boracyclic species (**109**), which hampered further applications.

**Scheme 30:**
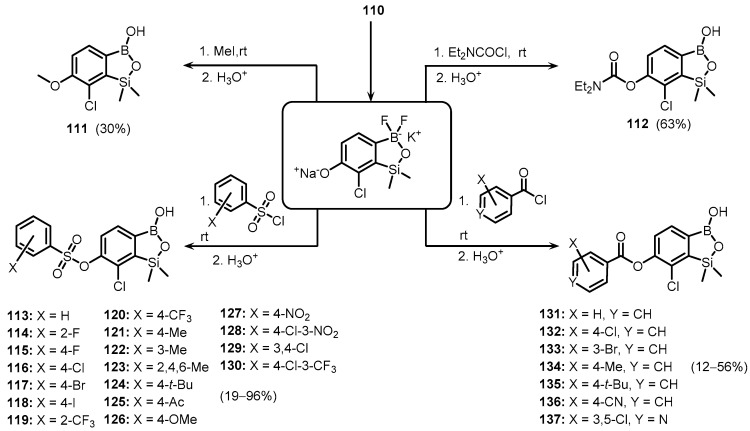
Synthesis of functionalized benzosiloxaboroles **111**–**137**.

The salt (**110**) was used as a phenolate *O*-nucleophile for reactions with MeI, Et_2_NCOCl, benzoyl, and benzenesulfonyl chlorides as electrophilic partners. The syntheses proceeded smoothly under mild conditions, giving rise to a series of functionalized benzosiloxaboroles (**111**–**137**), respectively (Scheme 30) [45].

**Scheme 31:**
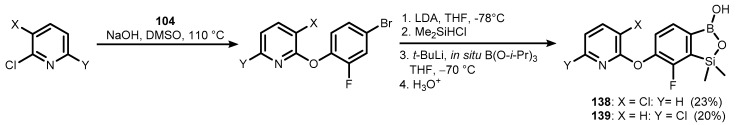
Synthesis of functionalized benzosiloxaboroles (**138**–**139**).

Structurally closely related 6-(chloropyridinyl-2-oxy)-7-fluorobenzosiloxaboroles (**138**–**139**) were also obtained using a standard protocol (Scheme 31) [45].

**Scheme 32:**
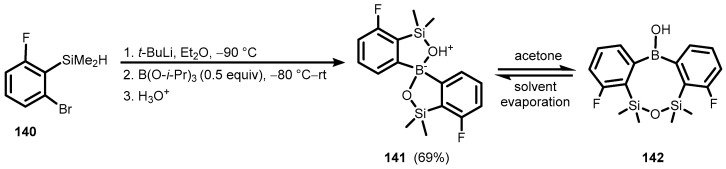
Synthesis and reversible rearrangement of spiro-borinate species **141**.

In addition, it is worth noting that Br-Li exchange in arylsilane (**140**), followed by treatment with B(O*i*-Pr)_3_ (0.5 equiv) gave selectively a unique borinate-type species (**141**) in ca. 70% yield (Scheme 32) [44]. The X-ray diffraction analysis revealed that its molecular structure features the spiro-arrangement of a central boron atom, linking two benzosiloxaborole systems. In crystal structure, discrete molecules form centrosymmetric dimers due to strong and symmetrical hydrogen bonds (O⋯O distance is only 2.432 Å). VT NMR analyses point to a dynamic character of **141** in CDCl_3_ solution. Remarkably, acetone-*d*_6_ (**141**) undergoes rearrangement to form 8-membered heterocyclic borinic acid (**142**) comprising the Si–O–Si linkage. However, the process is reversible because crystallization again afforded **141**, whilst prolonged exposure of the solution to air (>1 week) resulted in extensive degradation.

X-ray diffraction analyses of selected benzosiloxaboroles showed that their molecular structures are similar to those found for related benzoxaboroles. This is also true for supramolecular assembly, which is typically based on centrosymmetric H-bonded dimers [41,45]. Determination of the acidity of selected derivatives in water/methanol solution revealed that their p*K*_a_ values vary in a wide range (~4.0–8.0), depending on substitution pattern [43,45]. 1,1-Dimethyl-3-hydroxybenzosiloxaborole (**70**) is a stronger acid (p*K*_a_ = 7.9) than its benzoxaborole counterpart (p*K*_a_ = 8.3) [41]. Increased acidity of the boron atom in **70** arises apparently from its lower saturation by the endocyclic oxygen lone pairs owing to competition with distinctive Si-O bond conjugation (back-bonding effect). In addition, it was observed that the presence of more electron-withdrawing phenyl groups at the silicon atom increases the acidity in comparison to SiMe_2_ derivatives. In combination with perfluorination of the benzene ring, this resulted in the highest acidity of benzosiloxaborole (**77**) (p*K*_a_ = 4.2) among all of the studied derivatives. Notably, the acidity of borinate species (**141**) is even higher (p*K*_a_ = 3.5) [44] and very strongly enhanced with respect to the related simple 7-fluorobenzosiloxaborole (**72**) (p*K*_a_ = 7.2).

Selected functionalized 3-hydroxybenzosiloxaboroles emerged as novel small-molecule therapeutic agents. Simple halogenated derivatives (**72**–**80**) exhibit good antifungal activity with low Minimal Inhibitory Concentration (MIC) values (0.78–12.5 mg/L) for strains from *Candida* genus such as C. *albicans*, *C. tropicalis*, *C. krusei*, and *C. guilliermondii*. Thus, they can be regarded as effective silicon bioisosteres of related benzoxaboroles such as 5-fluorobenzoxaborole (Tavaborole), which has already been approved for the use against onychomycosis of the toenails [4].

Benzosiloxaboroles (**70**–**139**) were also screened as potential antibacterial agents against selected Gram-positive and Gram-negative strains. Overall, they showed at best a weak activity against Gram-negative bacilli, which was ascribed to the effective extrusion of used agents through bacterial walls by MDR efflux pumps. The highest increase in bacterial susceptibility to benzosiloxaboroles in the presence of efflux-pump inhibitor PAβN was observed for chloro derivatives (**79**-**80**) as MIC values decreased from 400 mg/L to 6.25 mg/L for *E. coli* and from 100–200 mg/L to 6.25 mg/L for *S. maltophilia* [43].

Benzosiloxaboroles show strongly varying activity against Gram-positive bacteria. The most promising results were obtained with respect to selected cocci strains such as *Staphylococcus aureus* (including MRSA strains), *S. epidermidis*, *Enterococcus faecalis*, and *E. faecium*, with MICs in the range of 6.25–50 mg/L in some cases. Interestingly, replacement of the fluoro substituent with the chloro one has the positive impact effect on the activity against Gram-positive cocci [43]. 6-Arylsulfonyloxy-7-chloro benzosiloxaboroles (**113**–**130**) showed high activity towards *S. aureus* species, including methicillin-resistant strains, with low MIC values in the range of 0.39–3.12 mg/L. Compounds **123**, **129**, and **130** also showed relatively high activity against other Gram-positive cocci such as *E. faecalis* and *E. faecium*, with MIC value of 6.25 mg/L. It should be noted that related 6-benzoyloxy derivatives (**131**–**137**) showed only moderate or weak activity against Gram-positive bacteria (MIC range = 12.5–400 mg/L), which indicates that benzenesulfonyl moiety is necessary to achieve high activity against studied cocci [45].

Benzosiloxaboroles (**70**–**96**) were also subjected to studies on inhibition of β-lactamases KPC- and pAmpC-produced by Gram-negative rods. Those enzymes provide multi-resistance to β-lactam antibiotics such as penicillins, cephalosporins, cephamycins, and carbapenems. Compounds **75, 76, 99** and, especially, 6-B(OH)_2_-substituted derivative (**95**) showed promising inhibitory activity. It should be noted that they were essentially inactive (MICs > 400 mg/L) against the studied Gram-negative strains when used alone but showed high activity when combined with meropenem. Molecular modelling studies confirmed strong inhibitory activity of those compounds with respect to KPC-2 carbapenemase [43].

The studies on antimicrobial activity of benzosiloxaboroles were complemented by evaluation of their cytotoxicity towards normal human lung fibroblasts MRC-5. It is worth noting that most of the studied compounds presented rather weak cytotoxicity or can be regarded as essentially non-toxic, which increases their potential in medicinal chemistry [43,45].

In addition, fluorinated 3-hydroxybenzosiloxaborole derivatives (**72**,**74**, and **76**) showed superior binding properties towards biologically-relevant diols in neutral pH aqueous conditions. Association constants *K*_s_ with dopamine, ribose, glucose, fructose, sorbitol, and adenosine and its monophosphate (AMP) were determined using the Springsteen and Wang method, with ARS as a fluorescent probe [41]. Later on, compounds **72**, **75**, **76**, **96**, and **102** were employed for the chemometric differential fluorescence-based sensing of saccharides such as glucose, fructose, ribose, sorbitol, lactose, and sucrose [46].

**Scheme 33:**
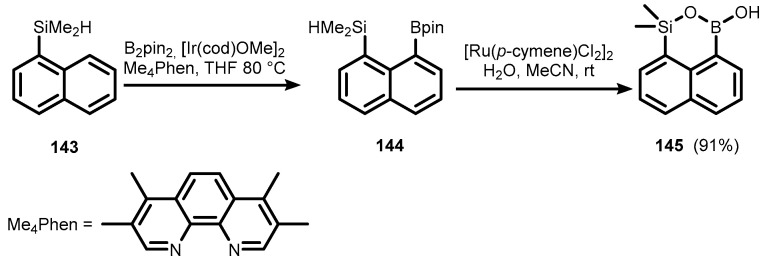
Synthesis of 1,1-dimethyloxasilaborinin-3-ol (**145**).

The synthesis and use of ring-expanded analogues of benzosiloxaboroles is currently at the initial stage of development. In 2018, Hartwig et al. obtained 1,1-dimethyloxasilaborinin-3-ol (**145**), comprising B-O-Si moiety bridging peri-naphthalene scaffold via Ir(I)-catalyzed CH-borylation of 1-(dimethylsilyl)naphthalene (**143**), followed by hydrolytic cleavage of intermediate pinacol boronate (**144**) in the presence of a Ru(II) catalyst. (Scheme 33) [47].

**Scheme 34:**
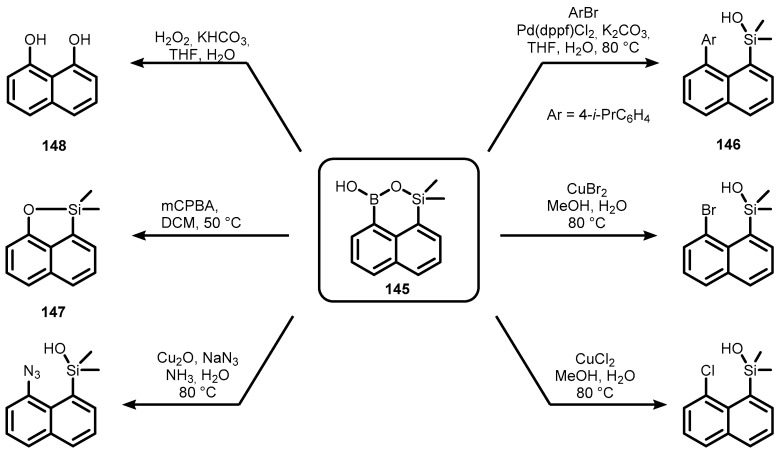
Transformations of **145** involving B-C bond activation.

Compound **145** was tested as a starting material for further transformations based on conversion of the B-C bond, including synthesis of a biaryl (**146**) by Suzuki–Miyaura cross coupling reaction, copper-mediated/catalyzed halogenation, and azide formation, as well as oxidation with meta-chloroperbenzoic acid (mCPBA), leading to the formation of naphthalene-fused oxasilole (**147**). Oxidation of both C-B and C-Si bonds was performed using an excess of H_2_O_2_ under basic conditions to give naphthalene-1,8-diol (**148**) (Scheme 34) [47].

**Scheme 35:**
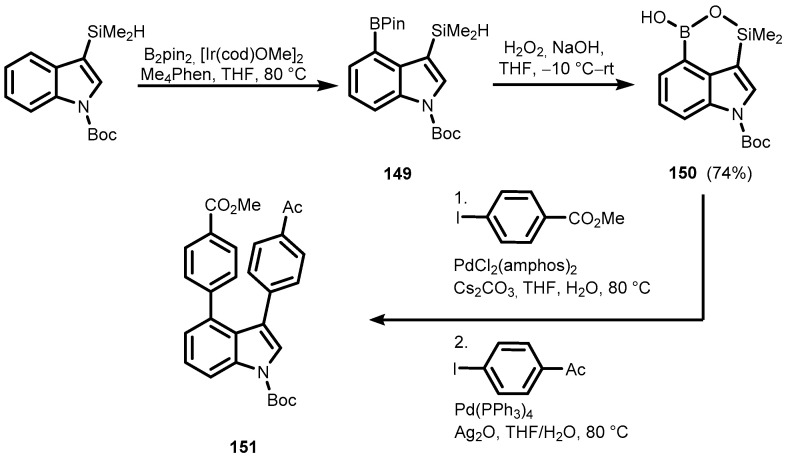
Synthesis and Pd-catalyzed double cross-coupling reaction of **150**.

Borylated indole (**149**) was obtained by a similar catalytic method reported for the synthesis of **143** (Scheme 35) [48]. A subsequent reaction with aq. H_2_O_2_ afforded indole-based oxasilaborininol (**150**) instead of an expected oxasilole derivative. It was converted to 3,4-diarylindole (**151**) by consecutive Suzuki–Miyaura and Hiyama cross-coupling reactions.

## 4. Benzoxaborole Congeners and Related Ring-Expanded Systems Comprising B-O-Sn Linkage

**Scheme 36:**
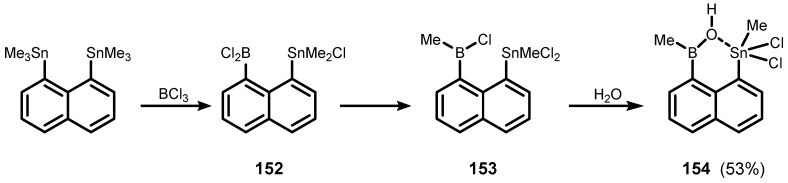
Synthesis of **154**.

There are only a few examples of heterocycles comprising B-O-Sn linkage fused with an aromatic core. The reaction of 1,8-bis(trimethylstannyl)naphthalene with BCl_3_ afforded 1-(chlorodimethylstannyl)-8-(dichloroboryl)naphthalene (**152**). It underwent chloride–methyl exchange at elevated temperature to give compound **153**, which was converted into the borinic acid derivative 1-(dichloromethylstannyl)-8-(hydroxymethylboryl)naphthalene (**154**). It was characterized by multinuclear NMR spectroscopy, and formation of a heterocyclic motif through dative B-O(H)→Sn interaction was confirmed by single crystal X-ray analysis (Scheme 36) [49].

**Scheme 37:**
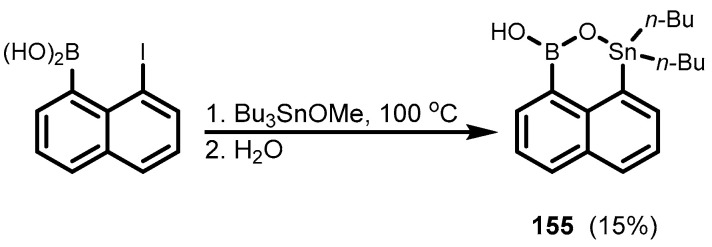
Synthesis of 1,1-dibutylnaphthooxastannaborinin-3-ol **155**.

Recently, 1,1-dibutylnaphthoxastannaborinin-3-ol (**155**), featuring covalent B-O-Sn linkage was obtained in low yields by the heating of 8-iodonaphthalene-1-boronic acid with an excess of Bu_3_SnOMe (Scheme 37) [50]. It was characterized in detail by single crystal X-ray diffraction analysis, which showed the formation of centrosymmetric H-bonded dimers.

**Scheme 38:**
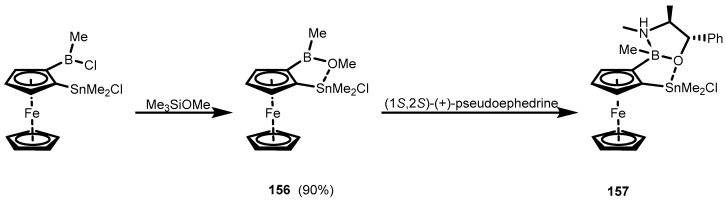
Synthesis of ferrocene derivatives **156**–**157** with fused heterocycles formed through dative B-O(Me)→Sn bond.

In addition, one can mention compound **156** with a ferrocene scaffold, where the formation of a heterocycle occurred through dative B-O(Me)→Sn interaction, resembling the situation observed in the naphthalene derivative (**154**). An extended complex (**157**) was isolated from the reaction of **156** with pseudoephedrine (Scheme 38) [51].

## 5. Benzoxaborole Aza Analogues and Related Ring-Expanded Systems

**Scheme 39:**
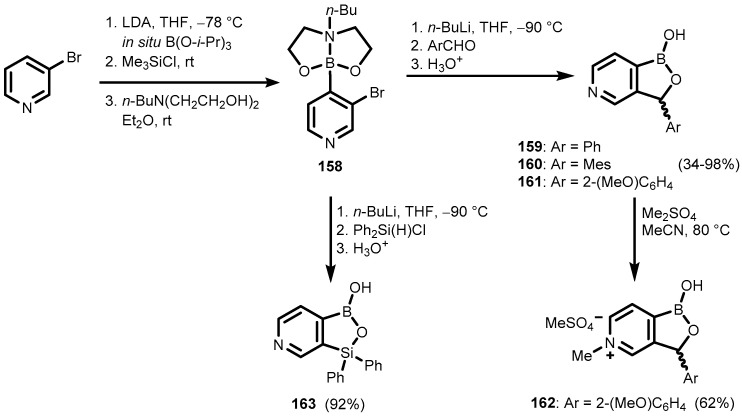
Synthesis of pyridoxaboroles **159**–**162** and pyridosiloxaborole **163**.

Nitrogen benzoxaborole congeners comprising a five-membered heterocycle with B-O-N linkage have not been described in the scientific literature so far. However, replacement of the benzene aromatic ring with a pyridine one gave rise to pyridoxaboroles. Their synthesis started with 3-bromopyridine, which was converted to 6-butyl-2-(3′-bromo-4′-pyridyl)-(N-B)-1,3,6,2-dioxazaborocan (**158**), followed by Br/Li exchange and the trapping of a resulting lithiated intermediate with selected benzaldehydes to give pyridoxaboroles **159**–**161**. Compound **161** was subjected to *N*-methylation, resulting in the cationic heterocycle (**162**). Pyridosiloxaborole (**163**) was obtained when Ph_2_Si(H)Cl was used as an electrophilic reagent (Scheme 39) [52].

**Scheme 40:**
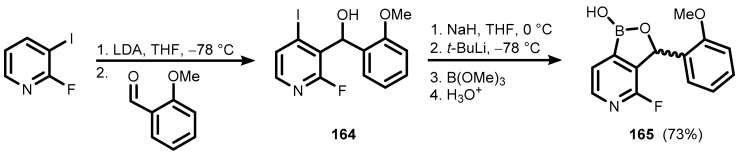
Synthesis of pyridoxaborole (**165**).

**Scheme 41:**
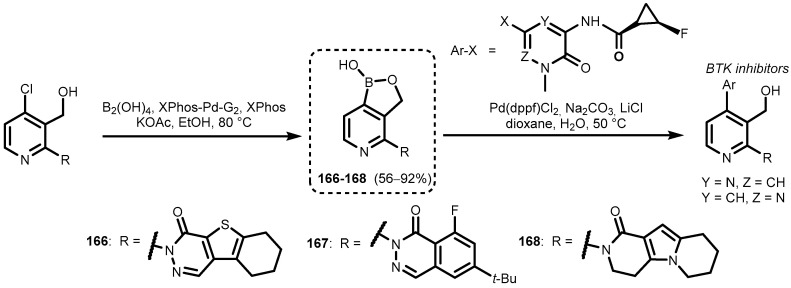
Synthesis and Suzuki–Miyaura cross-coupling reactions of pyridoxaboroles **166**–**168**.

In a different approach, 2-fluoro-3-iodopyridine was subjected to LDA-induced deprotonation with concomitant halogen-dance isomerization, followed by the trapping of an aryllithium with 2-methoxybenzaldehyde. The resultant carbinol (**164**) was protected as a sodium salt, which was subjected to I/Li exchange with *t*-BuLi and subsequent boronation with B(OMe)_3_ to give pyridoxaborole (**165**) (Scheme 40) [52]. In addition, pyridoxaboroles (**166**–**168**) were prepared recently and used in Suzuki–Miyaura cross-coupling reactions with selected halogenated *N*-methyl substituted pyrazinone and pyridazinone derivatives en route to novel Bruton’s tyrosine kinase (BTK) inhibitors, with the general structure shown in Scheme 41 [53].

Unlike structurally related benzoxaboroles, pyridoxaboroles are amphoteric compounds due to the presence of a basic nitrogen atom. ^11^B NMR spectroscopy data point to formation of zwitterionic species featuring the protonated nitrogen atom and anionic boronate moiety in a mixed MeOH/D_2_O solvent. The presence of the Lewis acidic boron and Lewis base N atom results in aggregation by means of N–B dative bonds, leading to the formation of a 1D coordination polymer of **161**. However, the introduction of a 2-fluoro substituent at the pyridine ring weakens N–B coordination and therefore H-bonded chains involving BOH groups and pyridine N atoms are formed in the case of **165** [52]. Thus, pyridoxaboroles can serve as self-complementary tectons for generation of molecular networks through N–B coordination and/or hydrogen bonds, where the assembly can be changed by tuning the donor properties of the pyridine N atom. The specific behaviour of pyridoxaboroles results from their amphoteric character, higher boron Lewis acidity as compared to pyridineboronic acids (or esters), and the presence of two competitive electron deficient sites, i.e., the boron and the BOH hydrogen atoms.

**Scheme 42:**
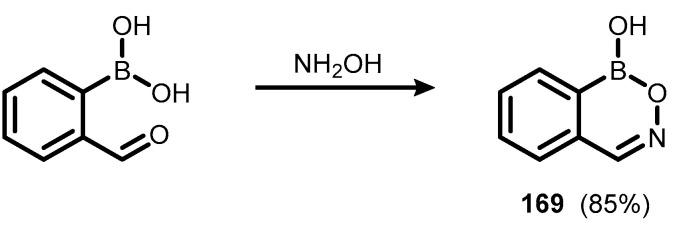
Synthesis of the parent benzoxazaborine **169**.

**Scheme 43:**
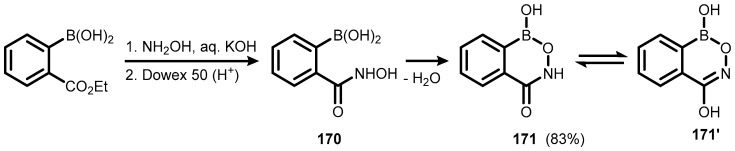
Synthesis of benzoxazaborininone **171**.

Ring-expanded benzoxaborole aza analogues featuring the 6-membered boracycle with B-O-N=C linkage, termed oxazaborines or nitrono arylboronate esters, have been reported already in the 1950s [54,55,56,57,58,59,60,61]. The parent benzoxazaborine (**169**) was obtained by condensation of 2-formylbenzeneboronic acid with hydroxylamine (Scheme 42) [54,56]. Renewed interest in this heterocycle emerged in 1997 thanks to Groziak et al., who undertook comprehensive research on boron-nitrogen planar heterocycles displaying nucleic acid-like hydrogen bonding motifs [62]. The analogous benzoxazaborininone (**171**) was obtained by aminolysis of ester group in (2-(ethoxycarbonyl)phenyl)boronic acid with hydroxylamine, followed by intramolecular condensation of boronic and hydroxamic groups in the intermediate (**170**), leading to a target planar 6-membered boron heterocycle ring. The X-ray diffraction analysis showed the presence of lactam tautomer (**171**)**,** rather than a lactim one (**171′**) (Scheme 43) [63].

**Scheme 44:**
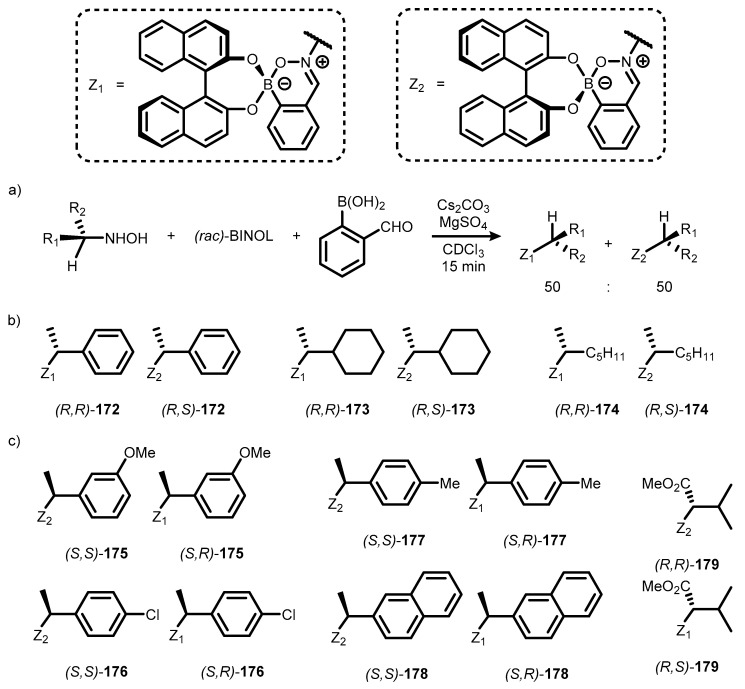
General procedure for preparation of mixtures of distareomeric nitrono-borane esters **170**–**177** (**a**); Complexes formed in reaction with (*R)*-hydroxylamines (**b**) and *(S)*-hydroxylamines (**c**).

Later, it was found that a simple three-component protocol involving the treatment of an appropriate chiral hydroxylamine with 2-formylphenylboronic acid and enantiopure BINOL leads to a mixture of diastereomeric nitrono-arylboronate esters, whose ratio can be determined by ^1^H NMR analysis and reflects the optical purity of a starting hydroxylamine (Scheme 44a). As a secondary effect, these studies gave rise to a series 4-substituted zwitterionic benzoxazaborine complexes (**172**–**179**), obtained as mixtures of diastereomers (Scheme 44b,c) [64].

**Scheme 45:**
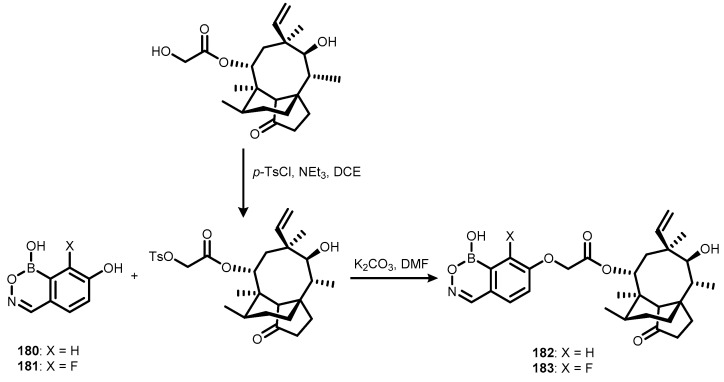
Synthesis of boracyclic pleuromutilin conjugates **182**–**183**.

Recently, two functionalized benzoxazaborine derivatives (**182**–**183**) were synthesized via the S_N_2 reactions of 7-hydroxybenzoxazaborines (**180**–**181**) and pleuromutilin tosylate (Scheme 45). It should be noted that the preparation of **180**–**181** was not reported. The pleuromutilin scaffold is a primary structural fragment of an important class of antibiotics. Thus, **182**–**183** were investigated as potential new anti-*Wolbachia* agents for the treatment of onchocerciasis and lymphatic filariasis [65]. Unlike benzoxaboroles, introduction of a fluorine atom did not improve antimicrobial potency, and only the EC_50_ values obtained for **180** (14.2 nM for Wolbachia infected C6/36 cells and 5.1 nM for Wolbachia infected LDW1 cells) are promising.

**Scheme 46:**
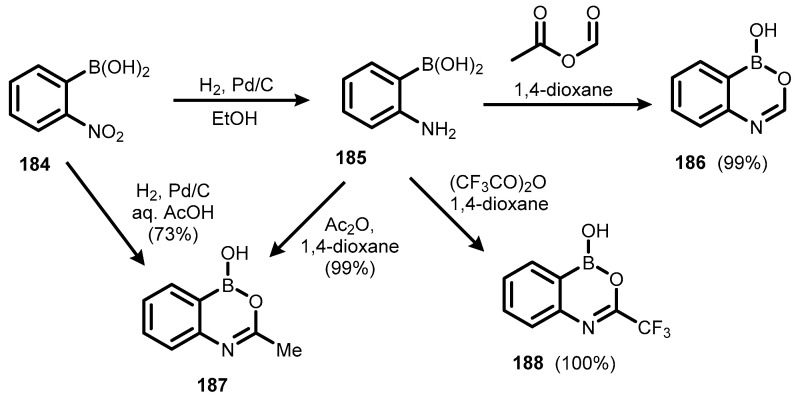
Synthesis of 1,2,4-benzoxazaborines **186**–**188**.

Another group of ring-expanded benzoxaborole aza analogues encompasses compounds with a 6-membered boracycle comprising B-O-C=N or B-O-C-N linkage, generally termed benzoxazaborines. They were reported for the first time over 60 years ago [66] but attracted increased interest only in the 1990s [67,68,69,70,71]. The parent benzoxazaborin (**186**) was synthesized by the reduction of (2-nitrophenyl)boronic acid (**184**) to (2-aminophenyl)boronic acid (**185**), which underwent condensation with acetic formic anhydride. 3-methyl and 3-trifluoromethyl substituted benzoxazaborines (**187**–**188**) were prepared analogously. Compound **187** was also synthesized from **184** via Parr hydrogenation in aqueous acetic acid, but this method was less efficient (Scheme 46) [68].

**Scheme 47:**
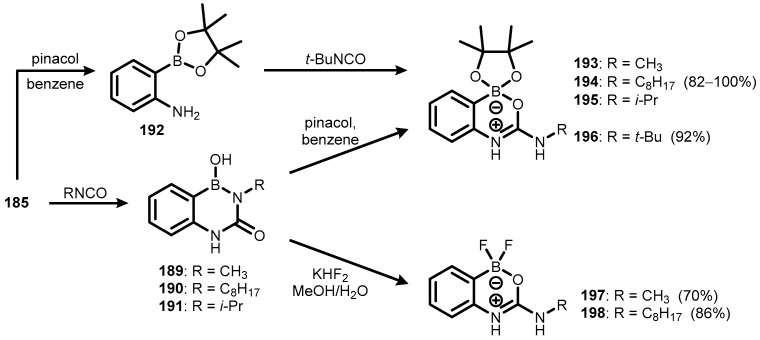
Synthesis of zwitterionic benzoxazaborines **193**–**198**.

**Scheme 48:**
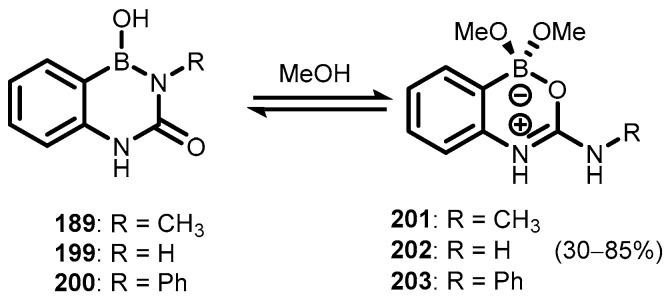
Reversible formation of zwitterionic benzoxazaborines **201**–**203**.

Zwitterionic benzoxazaborines featuring a four-coordinated boron atom (**193**–**198**) were also obtained. Thus, the treatment of **185** with selected alkyl isocyanates afforded respective diazaborine intermediates (**189**–**191**), which underwent condensation with pinacol to give **193**–**195**. In addition, the reactions of compounds **189** and **190** with KHF_2_ resulted in fluoro analogues **197** and **198**. In the case of tert-butyl derivative (**196**), diazaborine pinacol ester did not form, but the problem was overcome by conversion of **185** to pinacol ester (**192**), followed by treatment with *t*-BuNCO (Scheme 47) [69,70]. In the methanolic solution, diazaborines **189**, **199**, and **200** also undergo reversible transformation to zwitterionic oxazaborine systems (**201**–**203**, respectively) (Scheme 48) [71].

**Scheme 49:**
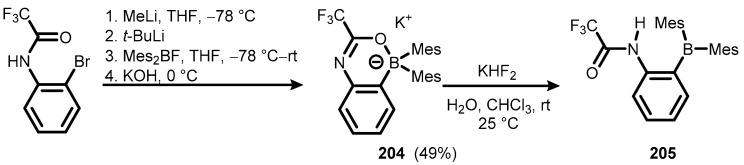
Synthesis of **205** via ionic benzoxazaborine intermediate **204**.

The potassium salt with oxazaborine anion (**204**) served as the intermediate in the synthesis, starting with *o*-bromotrifluoroacetanilide en route to functionalized triarylborane (**205**), which was expected to be an efficient fluoride receptor (Scheme 49) [72].

**Scheme 50:**
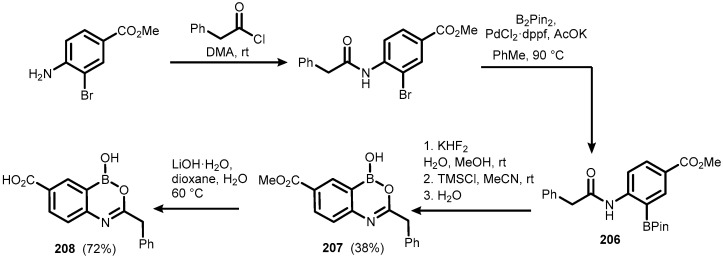
Synthesis of functionalized benzoxazaborines **207** and **208**.

**Scheme 51:**
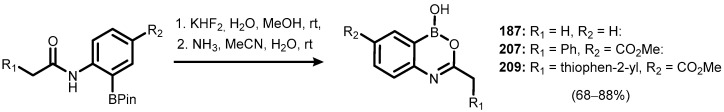
Synthesis of oxazaborine derivatives **187**, **207**, and **209**, utilizing the reaction of deprotection of *o*-amido pinacol phenylboronates via difluoroboranes.

More extended benzoxazaborines comprising substituents, both in the boracyclic ring as well as in the aromatic core, were reported in 2009. The synthesis of **207** and **208** started from methyl 4-amino-3-bromobenzoate, which was converted to an appropriate phenylacetamido derivative followed by the Miyaura borylation. Subsequent deprotection of boronate ester (**206**) gave compound **207,** which was additionally converted to benzoic acid derivative **208** (Scheme 50). Unfortunately, studies on the potential inhibitory activity of **207**–**208** against d,d-carboxypeptidase R39 did not give positive results [73]. In addition, an analogous variant of the deprotection reaction of pinacol boronate esters, utilizing milder NH_3_ base instead of LiOH in the second step, was elaborated in order to obtain compounds **187**, **207,** and **209** (Scheme 51) [74].

**Scheme 52:**
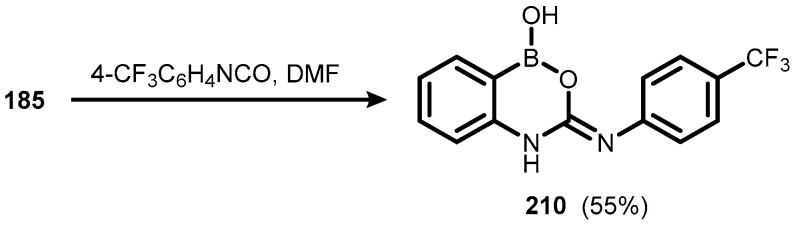
Synthesis of compound **210**.

Another functionalized derivative (**210**) was synthesized using **185** and 4-trifluoromethylphenyl isocyanate (Scheme 52). It was further subjected to investigation towards recognition of various guest molecules, especially warfare agents [75].

**Scheme 53:**
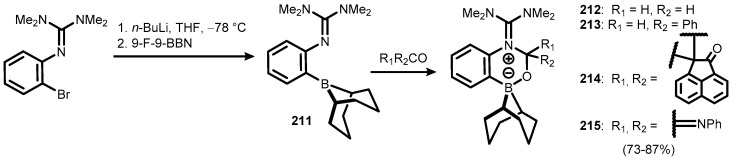
The use of the FLP effect for synthesis of boracycles **212**–**215** from ortho-borylated *N*-phenyltetramethylguanidine **211**.

Very recently, it was found that ortho-borylated *N*-phenyltetramethylguanidine (**211**) exhibits the Frustrated Lewis Pair (FLP) character, which enables formation of zwitterionic boracyclic adducts (**212**–**215**) through the insertion of carbonyl electrophiles such as H_2_CO, PhCHO, and PhNCO (Scheme 53) [76].

## 6. Benzophosphoxaboroles and Related Ring-Expanded Systems

**Scheme 54:**
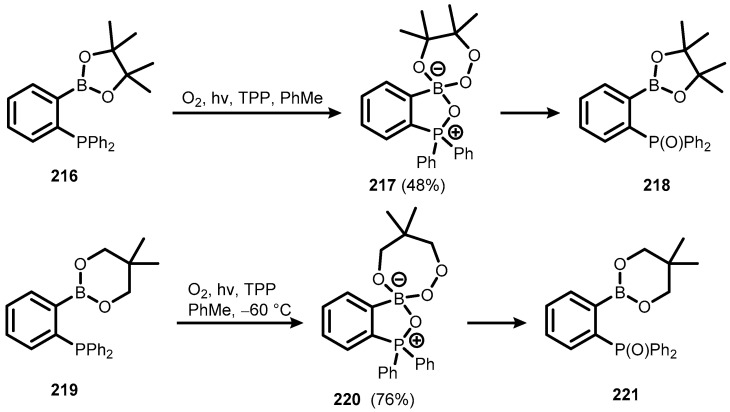
Synthesis and transformation of benzophosphoxaboroles **217** and **220**, comprising peroxoboronate motifs.

Benzophosphoxaboroles can be generally defined as heterocyclic systems comprising P-O-B linkage. There are a few examples of such compounds, which can be regarded as phosphine oxide systems stabilized through intramolecular P=O→B coordination but are also depicted as zwitterionic structures with P^+^-O-B^−^ linkage. The synthesis of benzophosphoxaboroles **217** and **220**, comprising cyclic peroxoboronate motifs, was achieved via ^1^O_2_ oxidation of ortho-(diphenylphosphino) phenyl boronates **216** and **219** and subsequent con-proportionation to ortho-boronated triphenylphosphine oxides (**218** and **221**) (Scheme 54) [77].

**Scheme 55:**
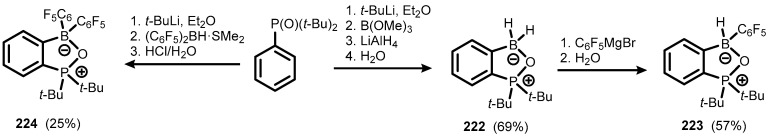
Synthesis of **222**–**224**.

Directed ortho-metalation of bis(tert-butyl)phosphine oxide with *t*-BuLi, followed by boronation with B(OMe)_3_ and reduction using LiAlH_4_, afforded a water-stable benzophosphoxaborole (**222**) featuring a P=O-BH_2_ moiety. It was converted to a mono-C_6_F_5_ derivative (**223**) through the nucleophilic attack of C_6_F_5_MgBr on the boron atom, followed by hydrolysis with a liberation of H_2_ (Scheme 55) [78]. A analogous bis(C_6_F_5_)-substituted derivative (**224**) was obtained by treatment of lithiated bis(*t*-butyl)phosphine oxide with (C_6_F_5_)_2_BH·SMe_2_, followed by acidic quenching of an intermediate ate complex. Compounds **222**–**224** were characterized by X-ray diffraction, which confirmed the formation of typical single B-O bonds (bond lengths in the range of 1.550–1.584 Å). Crystallographic data were consistent with ^11^B NMR chemical shifts, which confirmed the presence of the tetracoordinate boron atom. Furthermore, the ^31^P NMR resonances are deshielded by ca. 50 ppm compared to the starting phosphine oxide, which clearly reflects the strong O→B coordination.

**Scheme 56:**
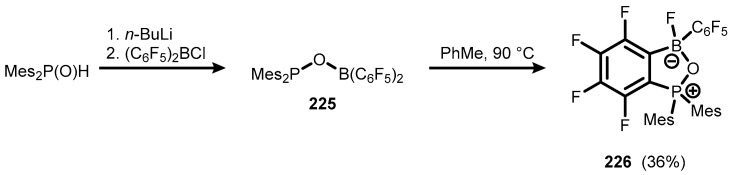
Synthesis of **226**.

Another benzophosphoxaborole derivative (**226**) was obtained in a two-step protocol starting with *O*-borylation of Mes_2_P(O)H, followed by thermally induced rearrangement of the intermediate phospinoxyborane (**225**) via intramolecular attack of the S_N_Ar nucleophilic P atom on the C-F bond, resulting in ring closure, with concomitant migration of fluoride to the boron atom (Scheme 56) [79].

**Scheme 57:**
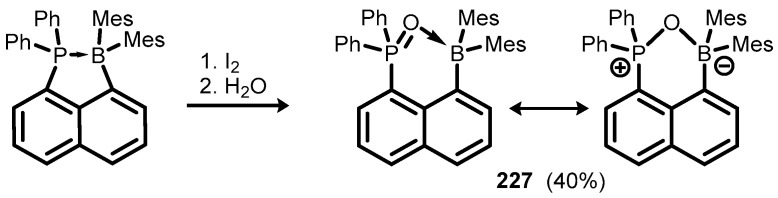
Synthesis of **227**.

Ring-expanded benzophosphoxaborole congeners are based on peri-substituted naphthalene and acenaphthene frameworks. Thus, 1-(dimesitylboryl)-8-(diphenylphosphino)naphthalene was subjected to oxidation of the P(III) centre with I_2_ followed by hydrolysis of ae P(V) hypercoordinated intermediate, producing phosphine oxide derivative **227**, which showed strong P=O→B coordination according to X-ray diffraction analysis; it was in agreement with ^11^B NMR chemical shift, pointing to the tetrahedral character of the boron atom. Additionally, ^31^P NMR resonance is significantly shifted to a lower field, which is characteristic for P=O systems coordinated to Lewis acid centres (Scheme 57) [80].

**Scheme 58:**
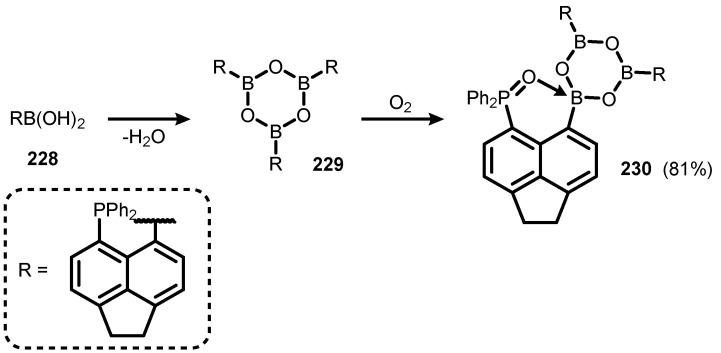
Synthesis of **230**.

Boroxine species **229**, resulting from dehydrative cyclotricondensation of 5-diphenylphosphinoacenaphth-6-yl boronic acid (**228**) was susceptible to partial oxidation in moist air, involving only one of three phosphorus atoms to give compound **230** (Scheme 58) [81].

**Scheme 59:**
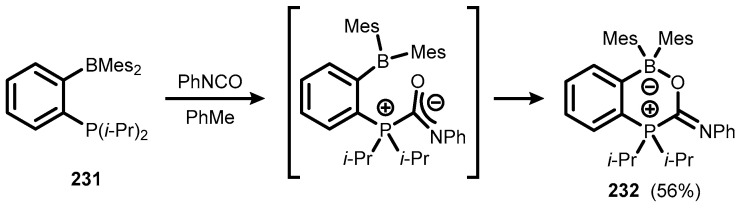
Synthesis of zwitterionic compound **232**.

A six-membered zwitterionic ring system (**232**), comprising an internal B-O bond separated from the phosphorus centre by a carbon atom was obtained by utilizing the distinctive FLP properties of an ambiphilic phosphinoborane *i*-Pr_2_P(*o*-C_6_H_4_)BMes_2_ **231** towards dipolar activation of phenyl isocyanate (Scheme 59) [82].

**Scheme 60:**
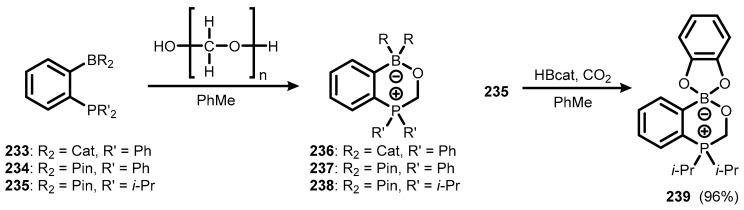
Synthesis of zwitterionic boron-phosphorus heterocycles **236**–**239**.

Similar six-membered zwitterionic heterocycles (**236**–**238**) are also simply formed by the treatment of ambiphilic ortho-phosphinyl pinacolato and catecholato arylboranes (**233**–**235**) with paraformaldehyde. Interestingly, it was found that CO_2_ can also be entrapped by **235** with the aid of CatBH as a reducing agent to give analogous system **239** (Scheme 60) [83].

**Scheme 61:**
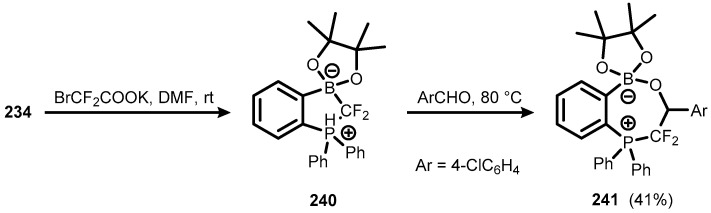
Synthesis of ring-expanded compound **241**.

The FLP behaviour of **234** was used for the trapping of in situ generated difluorocarbene to give a heterocyclic compound (**240**) featuring B-CF_2_-P moiety. The ring expansion in **240** occurred upon heating with 4-chlorobenzaldehyde through insertion of a carbonyl group, resulting in compound **241** (Scheme 61) [84].

**Scheme 62:**
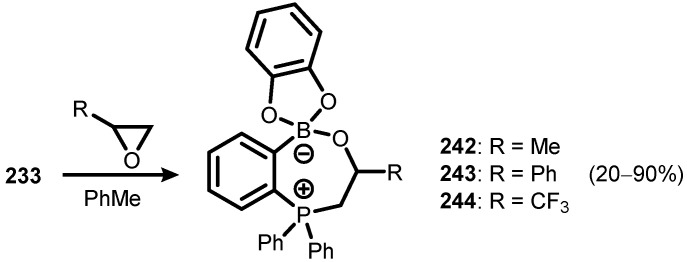
Synthesis of ring-expanded compounds **242**–**244** from insertion of epoxides with FLP species **233**.

Analogous 7-membered ring systems (**242**–**244**) were obtained by the ring opening of selected epoxides, mediated by the FLP compound **233** (Scheme 62) [85].

## 7. Benzoiodoxaboroles

**Scheme 63:**
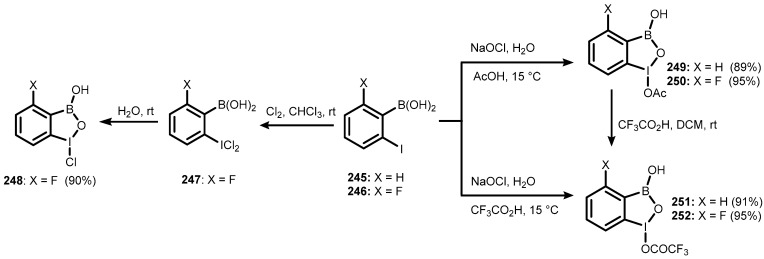
Synthesis of benzoiodoxaboroles **248**–**252**.

**Scheme 64:**
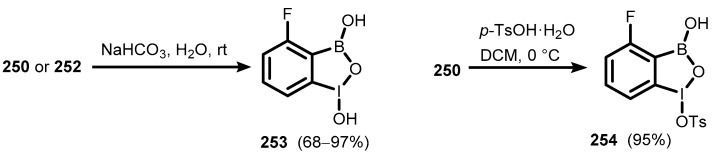
Preparation of benzoiodoxaboroles **253**–**254**.

Benzoiodoxaborole heterocycles comprising trivalent iodine, oxygen, and boron were only reported in 2011 [86]. In addition to being benzoxaborole congeners, those systems are also structurally related to benziodoxoles, representing an important part of the group of hypervalent iodine compounds extensively exploited in organic synthesis as highly selective and environmentally friendly oxidizing agents [87]. A 1-Chlorobenzoiodoxaborole derivative (**248**) was synthesized in a simple two-step process involving chlorination of 2-fluoro-6-iodophenylboronic acid (**246**), followed by the hydrolysis of intermediate **247**. Benzoiodoxaboroles (**248**–**251**), bearing acetoxy or trifluoroacetoxy substituent at the iodine atom were obtained by oxidation of iodophenylboronic acids (**245**–**246**) with bleach (~5% aqueous sodium hypochlorite) in acetic or trifluoroacetic acid. However, it was found that 1-trifluoroacetoxy derivatives **251** and **252** can be obtained in much higher yields (>90%) by the treatment of acetates **249** and **250** with an excess of CF_3_CO_2_H (Scheme 63) [86]. Hydrolysis of benzoiodoxaboroles **250** and **252**, carried out under mild basic conditions, afforded 1-hydroxy-4-fluorobenziodoxaborole (**253**). Treatment of **250** with *p*-TsOH H_2_O led to the formation of 1-tosyloxy-4-fluorobenzoiodoxaborole (**254**) (Scheme 64) [86].

**Scheme 65:**
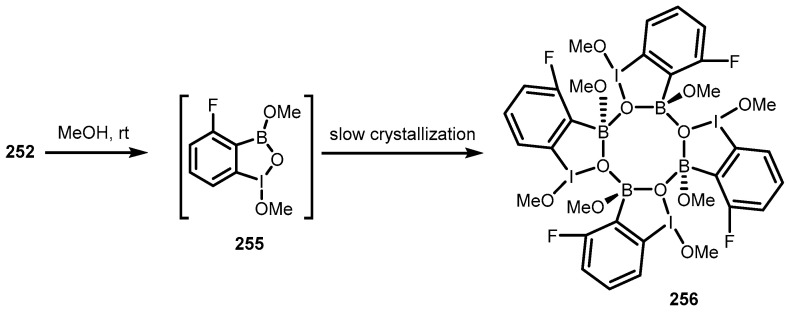
Formation of tetrameric benzoiodoxaborole aggregate **256**.

A slow (10 day) crystallization of **252** in methanol resulted in a unique tetrameric system (**256**) composed of four molecules of dimethoxy derivative **255**, assembled through dative interactions between boron and endocyclic oxygen atoms. Thus, the aggregate features the central 8-membered B_4_O_4_ ring (Scheme 65) [86].

**Scheme 66:**
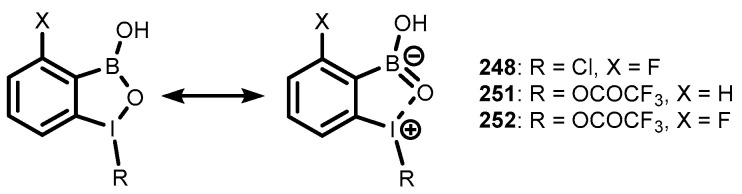
Resonance structures of a benzoiodoxaborole framework.

Crystallographic studies on benzoiodoxaboroles **248**, **251**, and **252**, comprising the trigonal-planar sp^2^ hybridized boron atom, indicates the presence of a planar five-membered iodoxaborole ring. The most noteworthy feature is the presence of unusually short endocyclic I–O bonds at 2.04–2.09 Å. In fact, they are the shortest ever observed for the five-membered iodine(III) heterocycles. Such a bond shortening, together with the heterocycle planarity, may point to some additional conjugation and possible aromatic character with the contribution of resonance structures shown in Scheme 66. However, DFT calculations of NICS(0) and NICS(1) indexes for 1-chloro- and 1-trifluoroacetoxy substituted benziodoxaboroles revealed low aromaticity of iodoxaborole heterocycle in comparison to typical aromatic rings [86].

Compounds **249**–**252** were tested as oxidizing agents in reactions with various organic substrates. However, they exhibit lower activity than 1-hydroxy- and 1-acetoxybenzoiodoxoles and do not oxidize alcohols, even when combined with a catalyst such as BF_3_ Et_2_O [86].

**Scheme 67:**
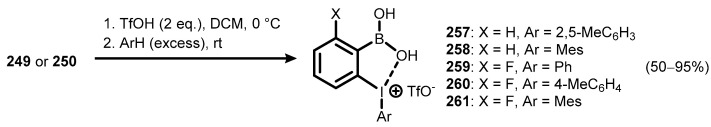
Synthesis of pseudocyclic ionic 1-arylbenziodoxaboroles **257**–**261**.

More recently, a new generation of pseudocyclic ionic benzoiodoxaboroles bearing various aryl substituents at the iodine atom was developed [88]. These new hypervalent iodine compounds were synthesized from 1-acetoxybenzoiodoxaboroles **249** and **250** and arenes by treatment with trifluoromethanesulfonic acid under mild conditions. Five derivatives (**257**–**261**) with various substitution patterns on the aryl group attached to hypervalent iodine were successfully obtained (Scheme 67). X-Ray analysis of **259** and **260** confirmed a pseudocyclic benziodoxaborole structure with rather short intramolecular interactions between the iodine and oxygen (I-O distance in the range of 2.698–2.717 Å).

**Scheme 68:**
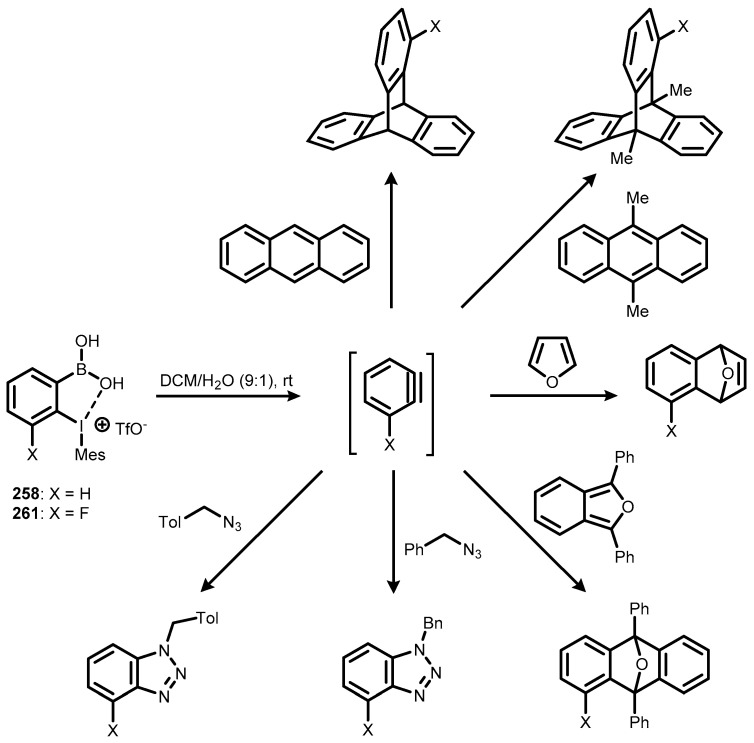
Aryne-trapping reactions involving pseudocyclic benzoiodoxaborole precursors **258** and **261**.

**Scheme 69:**
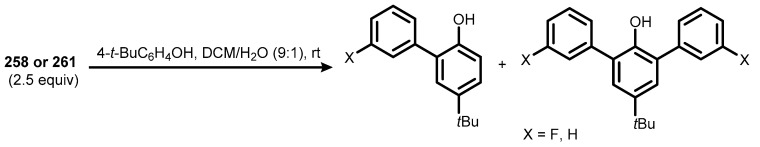
Arylation of 4-(*tert*-butyl)phenol with **258** and **261**.

Compounds **258** and **261** serve as new efficient benzyne generators, triggered by water in room temperature [88]. They were tested in reactions with various model substrates. The resultant aryne adducts were obtained in moderate to good yields under mild conditions, with water as the only activator of the reaction (Scheme 68). This is particularly important considering the fact that most of the benzyne precursors known to date require harsh or strongly basic conditions for the efficient generation of the benzyne intermediate. Moreover, further research showed that the new 1-arylbenzoiodoxaboroles could also serve as chemoselective arylating reagents towards the aromatic ring of tert-butyl phenol (Scheme 69) [88].

## 8. Conclusions

Heteroelement analogues of benzoxaboroles represent a diverse group of organoboron heterocycles. They exhibit strongly varying structural behaviour and different physicochemical properties. For example, benzosiloxaboroles can be regarded as close analogues of benzoxaboroles due to comparable stabilities of 5-membered oxaborole rings. In contrast, benzoxadiboroles are prone to hydrolytic ring opening. The Lewis acidity of the boron atom in presented systems also changes in a wide range, reflecting the strong effect of heteroatom substitution and other structural modifications with a special emphasis on fluorination of the aromatic ring. The varying properties open possibilities for many applications. Indeed, obtained systems have been used in organic synthesis as reagents or catalysts, fluorescence emitters for the construction of organic light-emitting diodes (OLEDs), and diol receptors, as well as potent antimicrobial agents. We hope that this review will stimulate further research in the area, which will result in the design of novel structures, including hitherto unknown heterocycles, e.g., comprising B-O-Ge or B-O-Sb linkage. Most importantly, the presented systems show significant practical potential, which should be exploited in future, especially for medicinal chemistry applications.

## Data Availability

Not applicable.

## References

[1] Liu C.T., Tomsho J.W., Benkovic S.J. (2014). The Unique Chemistry of Benzoxaboroles: Current and Emerging Applications in Biotechnology and Therapeutic Treatments. Bioorg. Med. Chem..

[2] Adamczyk-Woźniak A., Borys K.M., Sporzyński A. (2015). Recent Developments in the Chemistry and Biological Applications of Benzoxaboroles. Chem. Rev..

[3] Yang F., Zhu M., Zhang J., Zhou H. (2018). Synthesis of Biologically Active Boron-Containing Compounds. Med. Chem. Commun..

[4] Nocentini A., Supuran C.T., Winum J.-Y. (2018). Benzoxaborole Compounds for Therapeutic Uses: A Patent Review (2010–2018). Expert Opin. Ther. Pat..

[5] Kaufmann D.E., Boese R., Scheer A. (1994). 1,2-Bis(diisopropylamino)-1,2-dihydro-1,2-benzodiboret-ein erstes thermisch stabiles 1,2-Dihydro-1,2-diboret. Chem. Ber..

[6] Kaufmann D. (1987). Borylierung von Arylsilanen, II Synthese und Reaktionen silylierter Dihalogenphenylborane. Chem. Ber..

[7] Durka K., Jarzembska K.N., Kamiński R., Luliński S., Serwatowski J., Woźniak K. (2013). Nanotubular Hydrogen-Bonded Organic Framework Architecture of 1,2-Phenylenediboronic Acid Hosting Ice Clusters. Cryst. Growth Des..

[8] Durka K., Luliński S., Serwatowski J., Woźniak K. (2014). Influence of Fluorination and Boronic Group Synergy on the Acidity and Structural Behavior of *o*-Phenylenediboronic Acids. Organometallics.

[9] Adamczyk-Woźniak A., Cyrański M., Durka K., Gozdalik J., Klimentowska P., Rusiecki R., Sporzyński A., Zarzeczańska D. (2019). Structure and Properties of 1,3-Phenylenediboronic Acid: Combined Experimental and Theoretical Investigations. Crystals.

[10] Jarzembska K.N., Kamiński R., Durka K., Kubsik M., Nawara K., Witkowska E., Wiloch M., Luliński S., Waluk J., Głowacki I. (2017). New Class of Easily-Synthesisable and Modifiable Organic Materials for Applications in Luminescent Devices. Dye. Pigm..

[11] Jarzembska K.N., Kamiński R., Durka K., Kubsik M. (2017). Engineering of Solvatomorphs of the Luminescent Complex of *ortho* -Phenylenediboronic Acid and 8-Hydroxyquinoline. Cryst. Growth Des..

[12] Jarzembska K.N., Kamiński R., Durka K., Woźniak K. (2018). Ground-State Charge-Density Distribution in a Crystal of the Luminescent *Ortho*-Phenylenediboronic Acid Complex with 8-Hydroxyquinoline. J. Phys. Chem. A.

[13] Kutniewska S.E., Jarzembska K.N., Kamiński R., Stasyuk A.J., Gryko D.T., Cyrański M.K. (2018). Structural, Energetic and Spectroscopic Studies of New Luminescent Complexes Based on 2-(2′-Hydroxyphenyl)Imidazo[1,2-*a*]Pyridines and 1,2-Phenylenediboronic Acid. Acta Crystallogr. B Struct. Sci. Cryst. Eng. Mater..

[14] Williams V.C., Piers W.E., Clegg W., Elsegood M.R.J., Collins S., Marder T.B. (1999). New Bifunctional Perfluoroaryl Boranes. Synthesis and Reactivity of the *ortho*-Phenylene-Bridged Diboranes 1,2-[B(C_6_F_5_)_2_]_2_C_6_X_4_ (X = H, F). J. Am. Chem. Soc..

[15] Williams V.C., Irvine G.J., Piers W.E., Li Z., Collins S., Clegg W., Elsegood M.R.J., Marder T.B. (2000). Novel Trityl Activators with New Weakly Coordinating Anions Derived from C_6_F_4_-1,2-[B(C_6_F_5_)_2_]_2_: Synthesis, Structures, and Olefin Polymerization Behavior. Organometallics.

[16] Lewis S.P., Taylor N.J., Piers W.E., Collins S. (2003). Isobutene Polymerization Using a Chelating Diborane Co-Initiator. J. Am. Chem. Soc..

[17] Chai J., Lewis S.P., Collins S., Sciarone T.J.J., Henderson L.D., Chase P.A., Irvine G.J., Piers W.E., Elsegood M.R.J., Clegg W. (2007). Formation of Chelated Counteranions Using Lewis Acidic Diboranes: Relevance to Isobutene Polymerization. Organometallics.

[18] Henderson L.D., Piers W.E. (2007). Ion Pair Symmetrization in Metallocenium Cations Partnered with Diborane Derived Borate Counteranions. J. Organomet. Chem..

[19] Henderson L.D., Piers W.E., Irvine G.J., McDonald R. (2002). Anion Stability in Stannylium, Oxonium, and Silylium Salts of the Weakly Coordinating Anion [C_6_F_4_-1,2-{B(C_6_F_5_)_2_}_2_(μ-OCH_3_)]^−^. Organometallics.

[20] Lewis S.P., Chai J., Collins S., Sciarone T.J.J., Henderson L.D., Fan C., Parvez M., Piers W.E. (2009). Isobutene Polymerization Using Chelating Diboranes: Polymerization in Aqueous Suspension and Hydrocarbon Solution. Organometallics.

[21] Sgro M.J., Dömer J., Stephan D.W. (2012). Stoichiometric CO_2_ Reductions Using a Bis-Borane-Based Frustrated Lewis Pair. Chem. Commun..

[22] Letsinger R.L., Smith J.M., Gilpin J., MacLean D.B. (1965). Organoboron Compounds. XX. Chemistry of Some 1-Naphthaleneboronic Acids with Substituents in the 8-Position. J. Org. Chem..

[23] Katz H.E. (1985). Synthesis and Characterization of Novel 1*H*,3*H*-Naphth[1,8-cd][1,2,6]oxadiborins. J. Org. Chem..

[24] Scholz A.S., Massoth J.G., Bursch M., Mewes J.-M., Hetzke T., Wolf B., Bolte M., Lerner H.-W., Grimme S., Wagner M. (2020). BNB-Doped Phenalenyls: Modular Synthesis, Optoelectronic Properties, and One-Electron Reduction. J. Am. Chem. Soc..

[25] Rzepa H.S., Arkhipenko S., Wan E., Sabatini M.T., Karaluka V., Whiting A., Sheppard T.D. (2018). An Accessible Method for DFT Calculation of ^11^B NMR Shifts of Organoboron Compounds. J. Org. Chem..

[26] Watkinson M., Whiting A., McAuliffe C.A. (1994). Synthesis of a Bis-Manganese Water Splitting Complex. J. Chem. Soc. Chem. Commun..

[27] Li X., Han J.-W., Zhang Y.-X., Wong H.N.C. (2017). Palladium-Catalyzed Double Suzuki Reactions: Synthesis of Dibenzo[4,5:6,7]Cyclohepta[1,2,3-*de*]Naphthalenes. Asian J. Org. Chem..

[28] Kusukawa T., Mura R., Ooe M., Sumida R., Nakagawa A. (2021). Recognition of Carboxylic Acids and Phosphonic Acids Using 1,8-Diphenylnaphthalene-Based Diguanidine. Tetrahedron.

[29] Yang J., Horst M., Werby S.H., Cegelski L., Burns N.Z., Xia Y. (2020). Bicyclohexene-*Peri*-Naphthalenes: Scalable Synthesis, Diverse Functionalization, Efficient Polymerization, and Facile Mechanoactivation of Their Polymers. J. Am. Chem. Soc..

[30] IJpeij E.G., Beijer F.H., Arts H.J., Newton C., de Vries J.G., Gruter G.-J.M. (2002). A Suzuki Coupling Based Route to 2,2′-Bis(2-indenyl)biphenyl Derivatives. J. Org. Chem..

[31] Das A., Hübner A., Weber M., Bolte M., Lerner H.-W., Wagner M. (2011). 9-H-9-Borafluorene Dimethyl Sulfide Adduct: A Product of a Unique Ring-Contraction Reaction and a Useful Hydroboration Reagent. Chem. Commun..

[32] Shimada N., Hirata M., Koshizuka M., Ohse N., Kaito R., Makino K. (2019). Diboronic Acid Anhydrides as Effective Catalysts for the Hydroxy-Directed Dehydrative Amidation of Carboxylic Acids. Org. Lett..

[33] Shimada N., Takahashi N., Ohse N., Koshizuka M., Makino K. (2020). Synthesis of Weinreb Amides Using Diboronic Acid Anhydride-Catalyzed Dehydrative Amidation of Carboxylic Acids. Chem. Commun..

[34] Shimada N., Ohse N., Takahashi N., Urata S., Koshizuka M., Makino K. (2021). Direct Synthesis of *N*-Protected Serine- and Threonine-Derived Weinreb Amides via Diboronic Acid Anhydride-Catalyzed Dehydrative Amidation: Application to the Concise Synthesis of Garner’s Aldehyde. Synlett.

[35] Koshizuka M., Makino K., Shimada N. (2020). Diboronic Acid Anhydride-Catalyzed Direct Peptide Bond Formation Enabled by Hydroxy-Directed Dehydrative Condensation. Org. Lett..

[36] Radtke J., Schickedanz K., Bamberg M., Menduti L., Schollmeyer D., Bolte M., Lerner H.-W., Wagner M. (2019). Selective Access to Either a Doubly Boron-Doped Tetrabenzopentacene or an Oxadiborepin from the Same Precursor. Chem. Sci..

[37] Kawachi A., Zaima M., Tani A., Yamamoto Y. (2007). Dehydrogenative Condensation of (*o*-Borylphenyl)Hydrosilane with Alcohols and Amines. Chem. Lett..

[38] Kawachi A., Zaima M., Yamamoto Y. (2008). Intramolecular Reaction of Silanol and Triarylborane: Boron–Aryl Bond Cleavage and Formation of a Si–O–B Heterocyle. Organometallics.

[39] Shimizu T., Morisako S., Yamamoto Y., Kawachi A. (2020). Intramolecular Activation of C–O Bond by an *o*-Boryl Group in *o*-(Alkoxysilyl)(Diarylboryl)Benzenes. ACS Omega.

[40] Shimizu T., Kawachi A. (2020). Synthesis, Reactions, and Photophysical Properties of *o*-(Alkoxysilyl)(borafluorenyl)benzenes. J. Organomet. Chem..

[41] Brzozowska A., Ćwik P., Durka K., Kliś T., Laudy A.E., Luliński S., Serwatowski J., Tyski S., Urban M., Wróblewski W. (2015). Benzosiloxaboroles: Silicon Benzoxaborole Congeners with Improved Lewis Acidity, High Diol Affinity, and Potent Bioactivity. Organometallics.

[42] Czub M., Durka K., Luliński S., Łosiewicz J., Serwatowski J., Urban M., Woźniak K. (2017). Synthesis and Transformations of Functionalized Benzosiloxaboroles: Synthesis and Transformations of Functionalized Benzosiloxaboroles. Eur. J. Org. Chem..

[43] Durka K., Laudy A.E., Charzewski Ł., Urban M., Stępień K., Tyski S., Krzyśko K.A., Luliński S. (2019). Antimicrobial and KPC/AmpC Inhibitory Activity of Functionalized Benzosiloxaboroles. Eur. J. Med. Chem..

[44] Durka K., Urban M., Czub M., Dąbrowski M., Tomaszewski P., Luliński S. (2018). An Intramolecular *Ortho*-Assisted Activation of the Silicon–Hydrogen Bond in Arylsilanes: An Experimental and Theoretical Study. Dalton Trans..

[45] Pacholak P., Krajewska J., Wińska P., Dunikowska J., Gogowska U., Mierzejewska J., Durka K., Woźniak K., Laudy A.E., Luliński S. (2021). Development of Structurally Extended Benzosiloxaboroles–Synthesis and in Vitro Biological Evaluation. RSC Adv..

[46] Ćwik P., Ciosek-Skibińska P., Zabadaj M., Luliński S., Durka K., Wróblewski W. (2020). Differential Sensing of Saccharides Based on an Array of Fluorinated Benzosiloxaborole Receptors. Sensors.

[47] Su B., Hartwig J.F. (2018). Iridium-Catalyzed, Silyl-Directed, *Peri*-Borylation of C–H Bonds in Fused Polycyclic Arenes and Heteroarenes. Angew. Chem. Int. Ed..

[48] Sumida Y., Harada R., Sumida T., Hashizume D., Hosoya T. (2018). Hydrosilyl Group-Directed Iridium-Catalyzed *Peri*-Selective C–H Borylation of Ring-Fused (Hetero)Arenes. Chem. Lett..

[49] Schulte M., Gabbaï F.P. (2002). Synthesis of heteronuclear bifunctional Lewis acids by transmetalation of 1,8-bis(trimethylstannyl)naphthalene with BCl_3_. Can. J. Chem..

[50] Breitwieser K., Chen P. (2021). Crystal Structure of a 1,1-Dibutyl-1*H*,3*H*-naphtho[1,8-*Cd*][1,2,6]oxastannaborinin-3-ol. Acta Crystallogr. E Cryst. Commun..

[51] Boshra R., Venkatasubbaiah K., Doshi A., Jäkle F. (2009). Resolution of Planar-Chiral Ferrocenylborane Lewis Acids: The Impact of Steric Effects on the Stereoselective Binding of Ephedrine Derivatives. Organometallics.

[52] Steciuk I., Durka K., Gontarczyk K., Dąbrowski M., Luliński S., Woźniak K. (2015). Nitrogen–Boron Coordination versus OH⋯N Hydrogen Bonding in Pyridoxaboroles–Aza Analogues of Benzoxaboroles. Dalton Trans..

[53] Crawford J.J., Lee W., Johnson A.R., Delatorre K.J., Chen J., Eigenbrot C., Heidmann J., Kakiuchi-Kiyota S., Katewa A., Kiefer J.R. (2020). Stereochemical Differences in Fluorocyclopropyl Amides Enable Tuning of Btk Inhibition and Off-Target Activity. ACS Med. Chem. Lett..

[54] Snyder H.R., Reedy A.J., Lennarz W.J. (1958). Synthesis of Aromatic Boronic Acids. Aldehydo Boronic Acids and a Boronic Acid Analog of Tyrosine. J. Am. Chem. Soc..

[55] Dewar M.J.S., Dougherty R.C. (1962). Boron-Containing Analogs of Isoquinoline. J. Am. Chem. Soc..

[56] Dewar M.J.S., Dougherty R.C. (1964). New Heteroaromatic Compounds. XX. Derivatives of 4,3-Borazaroisoquinoline. J. Am. Chem. Soc..

[57] Dewar M.J.S., Jones R. (1967). New Heteroaromatic Compounds. XXV. Studies of Salt Formation in Boron Oxyacids by Boron-11 Nuclear Magnetic Resonance. J. Am. Chem. Soc..

[58] Davis F.A., Dewar M.J.S., Jones R. (1968). New Heteroaromatic Compounds. XXVII. Boron-11 Chemical Shifts of Some Heteroaromatic Boron Compounds. J. Am. Chem. Soc..

[59] Dougherty R.C. (1968). Mass Spectra of Heteroaromatic Boron Compounds. Tetrahedron.

[60] Dunn H.E., Catlin J.C., Snyder H.R. (1968). Arylboronic Acids. Imino Derivatives from *o*-Formylbenzeneboronic Acid. J. Org. Chem..

[61] Nanninga D., Kliegel W. (1983). Nitrone von 2-formylphenylboronsäureestern. J. Organomet. Chem..

[62] Groziak M.P., Chen L., Yi L., Robinson P.D. (1997). Planar Boron Heterocycles with Nucleic Acid-Like Hydrogen-Bonding Motifs. J. Am. Chem. Soc..

[63] Sarina E.A., Olmstead M.M., Nguyen D.N., Groziak M.P. (2013). 1-Hydroxy-1*H*-benzo[d][1,2,6]oxazaborinin-4(3*H*)-one. Acta Crystallogr C Cryst. Struct. Commun..

[64] Tickell D.A., Mahon M.F., Bull S.D., James T.D. (2013). A Simple Protocol for NMR Analysis of the Enantiomeric Purity of Chiral Hydroxylamines. Org. Lett..

[65] Jacobs R.T., Lunde C.S., Freund Y.R., Hernandez V., Li X., Xia Y., Carter D.S., Berry P.W., Halladay J., Rock F. (2019). Boron-Pleuromutilins as Anti-Wolbachia Agents with Potential for Treatment of Onchocerciasis and Lymphatic Filariasis. J. Med. Chem..

[66] Soloway A.H. (1960). Synthesis of Aromatic Diboronic Acids. J. Am. Chem. Soc..

[67] Groziak M.P. (2000). Boron Heterocycles as Platforms for Building New Bioactive Agents. Progress in Heterocyclic Chemistry.

[68] Groziak M.P., Ganguly A.D., Robinson P.D. (1994). Boron Heterocycles Bearing a Peripheral Resemblance to Naturally-Occurring Purines: Design, Syntheses, Structures, and Properties. J. Am. Chem. Soc..

[69] Hughes M.P., Shang M., Smith B.D. (1996). High Affinity Carboxylate Binding Using Neutral Urea-Based Receptors with Internal Lewis Acid Coordination. J. Org. Chem..

[70] Hughes M.P., Smith B.D. (1997). Enhanced Carboxylate Binding Using Urea and Amide-Based Receptors with Internal Lewis Acid Coordination: A Cooperative Polarization Effect. J. Org. Chem..

[71] Zhuo J.-C., Soloway A.H., Beeson J.C., Ji W., Barnum B.A., Rong F.-G., Tjarks W., Jordan G.T., Liu J., Shore S.G. (1999). Boron-Containing Heterocycles: Syntheses, Structures, and Properties of Benzoborauracils and a Benzoborauracil Nucleoside. J. Org. Chem..

[72] Hudnall T.W., Melaïmi M., Gabbaï F.P. (2006). Hybrid Lewis Acid/Hydrogen-Bond Donor Receptor for Fluoride. Org. Lett..

[73] Inglis S.R., Zervosen A., Woon E.C.Y., Gerards T., Teller N., Fischer D.S., Luxen A., Schofield C.J. (2009). Synthesis and Evaluation of 3-(Dihydroxyboryl)Benzoic Acids as d,d-Carboxypeptidase R39 Inhibitors. J. Med. Chem..

[74] Inglis S.R., Woon E.C.Y., Thompson A.L., Schofield C.J. (2010). Observations on the Deprotection of Pinanediol and Pinacol Boronate Esters via Fluorinated Intermediates. J. Org. Chem..

[75] Hiscock J.R., Wells N.J., Ede J.A., Gale P.A., Sambrook M.R. (2016). Biasing Hydrogen Bond Donating Host Systems towards Chemical Warfare Agent Recognition. Org. Biomol. Chem..

[76] Manankandayalage C., Unruh D.K., Krempner C. (2021). Small Molecule Activation with Intramolecular “Inverse” Frustrated Lewis Pairs. Chem. Eur. J..

[77] Porcel S., Bouhadir G., Saffon N., Maron L., Bourissou D. (2010). Reaction of Singlet Dioxygen with Phosphine-Borane Derivatives: From Transient Phosphine Peroxides to Crystalline Peroxoboronates. Angew. Chem. Int. Ed..

[78] Breunig J.M., Lehmann F., Bolte M., Lerner H.-W., Wagner M. (2014). Synthesis and Reactivity of *o*-Phosphane Oxide Substituted Aryl(Hydro)Borates and Aryl(Hydro)Boranes. Organometallics.

[79] Wang Y., Li Z.H., Wang H. (2018). Synthesis of an Oxygen-Linked Germinal Frustrated Lewis Pair and Its Application in Small Molecule Activation. RSC Adv..

[80] Li Y.-F., Kang Y., Ko S.-B., Rao Y., Sauriol F., Wang S. (2013). Highly Congested Donor–Acceptor P–B Compound: Synthesis and Properties of a BMes_2_-and a PPh_2_-functionalized 1,8-naphthalene. Organometallics.

[81] Furan S., Hupf E., Lork E., Beckmann J. (2021). Synthesis and Structure of 5-Diphenylphosphino- Acenaphth-6-yl Boronic Acid, Related Dialkyl Esters and Boroxine Rings. Z. Anorg. Allg. Chem..

[82] Moebs-Sanchez S., Bouhadir G., Saffon N., Maron L., Bourissou D. (2008). Tracking Reactive Intermediates in Phosphine-Promoted Reactions with Ambiphilic Phosphino-Boranes. Chem. Commun..

[83] Declercq R., Bouhadir G., Bourissou D., Légaré M.-A., Courtemanche M.-A., Nahi K.S., Bouchard N., Fontaine F.-G., Maron L. (2015). Hydroboration of Carbon Dioxide Using Ambiphilic Phosphine–Borane Catalysts: On the Role of the Formaldehyde Adduct. ACS Catal..

[84] Smirnov V.O., Volodin A.D., Korlyukov A.A., Dilman A.D. (2020). Trapping of Difluorocarbene by Frustrated Lewis Pairs. Angew. Chem. Int. Ed..

[85] Krachko T., Nicolas E., Ehlers A.W., Nieger M., Slootweg J.C. (2018). Ring-Opening of Epoxides Mediated by Frustrated Lewis Pairs. Chem. Eur. J..

[86] Nemykin V.N., Maskaev A.V., Geraskina M.R., Yusubov M.S., Zhdankin V.V. (2011). Preparation and X-Ray Crystal Study of Benziodoxaborole Derivatives: New Hypervalent Iodine Heterocycles. Inorg. Chem..

[87] Yoshimura A., Zhdankin V.V. (2016). Advances in Synthetic Applications of Hypervalent Iodine Compounds. Chem. Rev..

[88] Yoshimura A., Fuchs J.M., Middleton K.R., Maskaev A.V., Rohde G.T., Saito A., Postnikov P.S., Yusubov M.S., Nemykin V.N., Zhdankin V.V. (2017). Pseudocyclic Arylbenziodoxaboroles: Efficient Benzyne Precursors Triggered by Water at Room Temperature. Chem. Eur. J..

